# Heat Shock Protein 90 Chaperone Regulates the E3 Ubiquitin-Ligase Hakai Protein Stability

**DOI:** 10.3390/cancers12010215

**Published:** 2020-01-15

**Authors:** Andrea Díaz-Díaz, Daniel Roca-Lema, Alba Casas-Pais, Gabriela Romay, Giovanni Colombo, Ángel Concha, Begoña Graña, Angélica Figueroa

**Affiliations:** 1 Epithelial Plasticity and Metastasis Group, Instituto de Investigación Biomédica de A Coruña (INIBIC), Complexo Hospitalario Universitario de A Coruña (CHUAC), Sergas, Universidade da Coruña (UDC), 15006 A Coruña, Spain; andrea.diaz.diaz@sergas.es (A.D.-D.); Daniel.roca.lema@sergas.es (D.R.-L.); alba.casas.pais@sergas.es (A.C.-P.); Gabriela.Romay.Cousido@sergas.es (G.R.); 2 Istituto per la Ricerca e l’Innovazione Biomedica (IRIB)—CNR di Palermo, Via Ugo La Malfa 153, 90146 Palermo, Italy; giovanni.colombo@community.unipa.it; 3 Pathology Department and A Coruña Biobank from Instituto de Investigación Biomédica de A Coruña (INIBIC), Complexo Hospitalario Universitario de A Coruña (CHUAC), Sergas, Universidade da Coruña (UDC), 15006 A Coruña, Spain; angel.concha.lopez@sergas.es; 4 Clinical Oncology Group, Instituto de Investigación Biomédica de A Coruña (INIBIC), Complexo Hospitalario Universitario de A Coruña (CHUAC), Sergas, Universidade da Coruña (UDC), 15006 A Coruña, Spain; Begona.Grana.Suarez@sergas.es

**Keywords:** Hsp90 chaperone, E3 ubiquitin-ligase Hakai, targeted therapy, colon cancer

## Abstract

The E3 ubiquitin-ligase Hakai binds to several tyrosine-phosphorylated Src substrates, including the hallmark of the epithelial-to-mesenchymal transition E-cadherin, and signals for degradation of its specific targets. Hakai is highly expressed in several human cancers, including colon cancer, and is considered as a drug target for cancer therapy. Here, we report a link between Hakai and the heat shock protein 90 (Hsp90) chaperone complex. Hsp90 participates in the correct folding of its client proteins, allowing them to maintain their stability and activity. Hsp90 inhibitors specifically interfere with the association with its Hsp90 client proteins, and exhibit potent anti-cancer properties. By immunoprecipitation, we present evidence that Hakai interacts with Hsp90 chaperone complex in several epithelial cells and demonstrate that is a novel Hsp90 client protein. Interestingly, by overexpressing and knocking-down experiments with Hakai, we identified Annexin A2 as a Hakai-regulated protein. Pharmacological inhibition of Hsp90 with geldanamycin results in the degradation of Hakai in a lysosome-dependent manner. Interestingly, geldanamycin-induced Hakai degradation is accompanied by an increased expression of E-cadherin and Annexin A2. We also show that geldanamycin suppresses cell motility at least in part through its action on Hakai expression. Taken together, our results identify Hakai as a novel Hsp90 client protein and shed light on the regulation of Hakai stability. Our results open the possibility to the potential use of Hsp90 inhibitors for colorectal cancer therapy through its action on Hakai client protein of Hsp90.

## 1. Introduction

Hsp90 (90-KDa heat shock protein) is a molecular chaperone involved in the correct folding of a selected group of proteins, named as client proteins, allowing them to maintain their proper conformation and the preservation of their activity [1]. The regulation of Hsp90 client proteins not only plays a crucial role in several cellular processes, such as cell cycle control, apoptosis and cell survival, but also contributes to the development of pathological conditions, such as neurodegenerative and infectious disease or cancer [2]. Indeed, many client proteins of Hsp90 are oncogenes, mutated or overexpressed, which are important for tumor malignant progression [3]. The activation of Hsp90 client proteins occurs in an ATP-dependent manner [4]. The small-molecule inhibitor geldanamycin (GA), a benzoquinone ansamycin antibiotic, occupies the ATP binding-pocket of Hsp90, specifically interfering on the association with its client proteins. The general described dissociation between Hsp90 and their client proteins results on the degradation of the clients by the ubiquitin-dependent proteasome pathway [5,6,7]. However, new findings provide evidence of an autophagy-dependent mechanism [8]. A quantitative high-throughput analysis of the Hsp90-client proteins interactions reveals that only 7% are transcription factors, 30% are human ubiquitin-ligases and 60% kinases [1,9]. Therefore, it is reasonable to assume that additional E3 ubiquitin-ligases that associates to Hsp90 await to be elucidated.

Hakai is a new class of the three families of RING-finger type E3 ubiquitin-ligases that contains a novel domain called HYB (Hakai pTyr-binding) whereby it interacts with the tyrosine-phosphorylated substrates inducing their ubiquitination and degradation [10,11]. So far, the best-characterized substrate for Hakai is E-cadherin. Hakai interacts with the Src tyrosine-phosphorylated E-cadherin, inducing its ubiquitination and degradation, which in turn causes the alteration of cell–cell contacts [12,13]. E-cadherin is the best-characterized member of the adherens junctions and its loss at cell–cell adhesions is a well stablished hallmark of the epithelial-to-mesenchymal transition during tumor progression and metastasis [14,15]. Hakai is not only involved in the regulation of cell–cell adhesions but also in regulating cell proliferation, cell motility and invasion [16,17]. Importantly, Hakai is highly expressed in colon and lung cancer compared to human healthy tissues [18,19,20]. Additional target proteins for Hakai, such as Cortactin or DOK1, have been identified, however, the functional significance is still unknown [10]. Recently, we employed an iTRAQ approach and found differentially regulated proteins in epithelial MDCK cell lines stably expressing Hakai compared to non-transformed MDCK cells. Interestingly, several membrane-binding proteins, including Annexin A2, were confirmed to be decreased in Hakai-overexpressing cells while other proteins, such as Hsp90 chaperone, were slightly increased [21].

In this paper, we were interested to understand the meaning of the link between the E3 ubiquitin-ligases Hakai and Hsp90 chaperone complex. Here, we demonstrate that the E3 ubiquitin-ligase is a novel client protein for Hsp90. Pharmacological inhibition of Hsp90 with geldanamycin disrupts this interaction, and induces Hakai degradation via lysosome, which in consequence is accompanied by an increased expression of E-cadherin and Annexin A2. Importantly, geldanamycin reduces Hakai-induced cell migration, further underscoring the possible impact of Hsp90 inhibitors in tumor progression by its action on Hakai stability.

## 2. Results

### 2.1. Hsp90 Chaperone Interacts with Hakai

**Figure 1:**
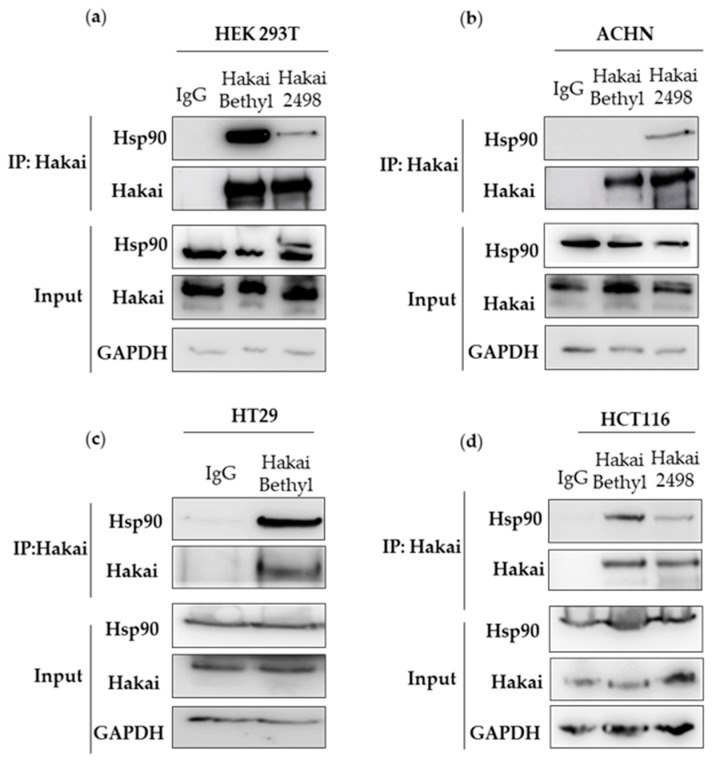
Hakai interacts with heat shock protein 90 (Hsp90): Co-immunoprecipitation of Hakai and Hsp90 was performed in different cell lines: (**a**) HEK293T. (**b**) ACHN. (**c**) HT29. (**d**) HCT116. Hakai immunoprecipitation was performed by employing two different rabbit polyclonal Hakai antibodies indicated in material an methods: one from Bethyl (Bethyl Ab) and one kindly provided by Dr. Fujita (Hakai-2498). GAPDH signal was used as protein loading control.

In a proteomic study conducted for the search of interacting proteins with Hsp90, Taipale and collaborators showed that, far from expected, more than 100 proteins were E3 ubiquitin-ligases [1,9]. However, only few of these interactions have been demonstrated, such as the interaction with the E3 ubiquitin ligases Cullin-5 or CHIP, [22,23]. Given that we have previously shown a regulation of Hsp70 and Hsp90 chaperones in Hakai stably expressing MDCK epithelial cells compared to non-transformed epithelial cells [21], we decided to evaluate whether Hakai could be among the E3 ubiquitin ligases interacting proteins with these chaperones. In a first experiment, we immunoprecipitated endogenous Hakai using several different cell lines, including HEK293T, ACHN, HT29 and HCT116 (Figure 1, Supplementary Figure S4). Co-immunoprecipitation of Hakai and Hsp90, but not Hsp70 was detected in all four cell lines tested.

Moreover, the analysis of Hakai interactome in HCT116, by employing endogenous Hakai immunoprecipitation following by nano-flow liquid chromatography (LC) coupled to a triple TOF Mass Spectrometer, also identified Hakai and Hsp90 proteins in Hakai immunoprecipitation samples compared to IgG control sample, further confirming the interaction between these two proteins. However, no effect on Hsp90 protein levels was detected when Hakai was transiently transfected in HEK293T cells in a concentration-dependent manner (Supplementary Figures S1a and S10) neither in a time-dependent manner (Figure S1b, Supplementary Figure S10). Similarly, we neither detected any effect on Hakai expression by transiently transfecting increasing amounts of Hsp90 (Supplementary Figures S1c and S10). Since we previously demonstrated that Hakai is highly expressed in human colon cancer tissues compared to healthy colon tissues, we also analyzed the possible co-localization by using HT29, LoVo and HCT116 colon cancer cell lines. Endogenous Hakai is highly detected in the nucleus and less intense signal is observed in the cytoplasm, where it is described to exert its E3 ubiquitin-ligase activity. Hsp90 is clearly localized throughout the whole cytoplasm where co-localization with Hakai is slightly detected in HT29 and Lovo cells, with an enriched signal detected in perinuclear areas (Supplementary Figure S2).

### 2.2. Interaction between Hsp90, Hakai and Annexin A2

**Figure 2:**
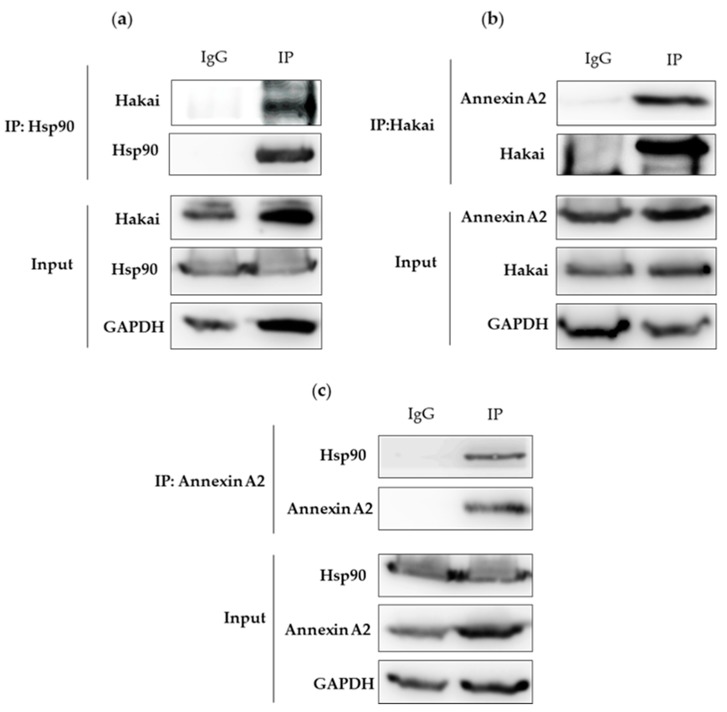
Hakai, Annexin A2, and Hsp90 interact with other: The whole cell extracts of HCT116 cell line cells were prepared and subjected to a co-immunoprecipitation assay. Subsequent western-blotting analysis was performed by using Hakai, Annexin A2, or Hsp90 antibodies (**a**) Hakai is specifically detected in anti-Hsp90 immunoprecipitates. (**b**) Annexin A2 is specifically detected in anti-Hakai immunoprecipitates. (**c**) Hsp90 is specifically detected in anti-Annexin A2 immunoprecipitates. GAPDH signal was used as protein loading control.

It is generally reported that chaperone-interacting E3 ubiquitin-ligases induce the degradation of the chaperone client proteins that have not been correctly folded [22,23,24]. Therefore, it is reasonable to assume that additional E3 ubiquitin-ligases are involved in the degradation of HSP90 clients. Given the interaction confirmed between Hakai and Hsp90, our findings led us to ask whether Hakai may also participate in the regulation of other Hsp90 client proteins. Annexin A2 was one of the proteins detected in a proteomic study that turned out to be downregulated in Hakai-overexpressing MDCK cells compared to non-transformed MDCK epithelial cells [21]. In addition, it has been reported that Annexin A2 co-immunoprecipitates with Hsp90 [25]. Annexin A2 is a calcium-binding protein reported to be implicated in membrane and vesicle trafficking [26]. Considering these premises, we further analyze the possible interaction between these three proteins. Endogenous interaction of Hsp90 with Hakai and Annexin A2 was confirmed in HCT116 cells. Immunoprecipitation of endogenous Hakai co-precipitated with endogenous Hsp90 (Figure 2a, Supplementary Figure S5). Endogenous Annexin A2 also co-precipitated with endogenous Hakai (Figure 2b, Supplementary Figure S5). Conversely, immunoprecipitation of Annexin A2 also co-precipitated with endogenous Hsp90 (Figure 2c, Supplementary Figure S5). These results confirm that Hakai, Hsp90 and Annexin A2 form an interacting protein complex, either mediated by a direct or indirect interaction.

### 2.3. Hakai Regulates Annexin A2 Protein Expression

**Figure 3:**
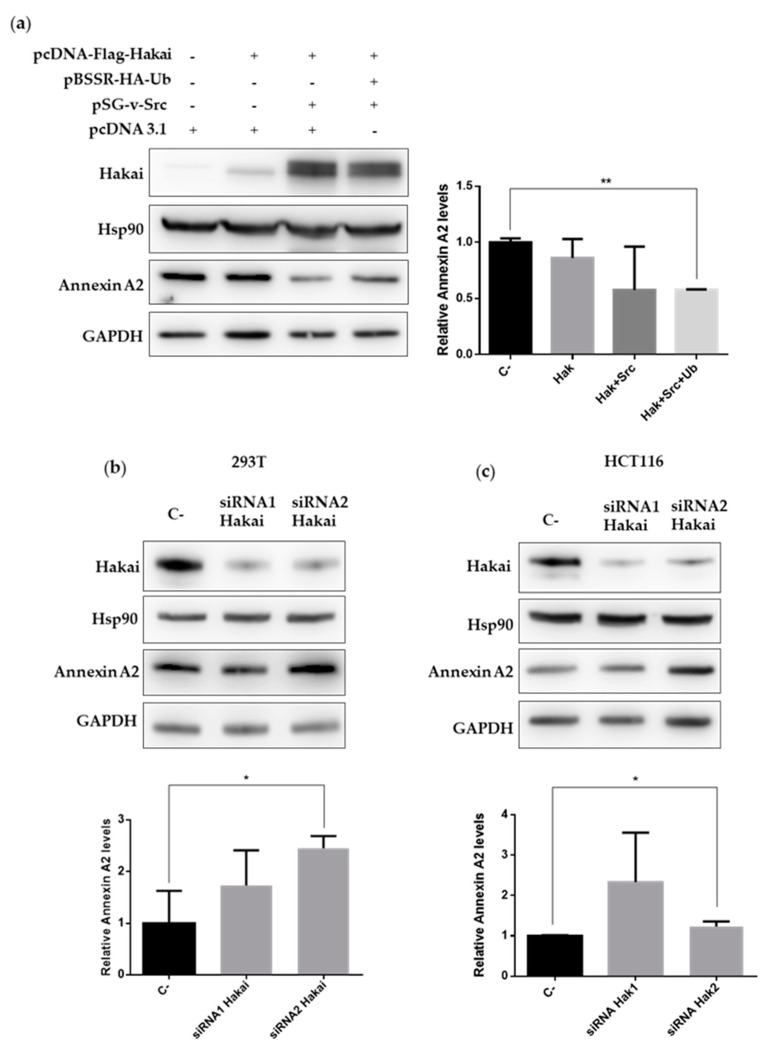
Hakai regulates Annexin A2 expression levels: (**a**) HEK293T cells were transiently transfected with pcDNA-Flag-Hakai (4 µg), pBSSR-HA-ubiquitin (3 µg) and pSG-v-Src (3 µg) plasmids for 48 h. Cells were harvested and the levels of endogenous Annexin A2 levels and Hsp90 were determined by western blot analysis (left panel) and quantified by densitometry (right panel). The effect of the indicated transfected siRNA-Hakai on Annexin A2 and Hsp90 levels were tested in 293T cells (**b**) and HCT116 (**c**). Whole-cell lysates were subjected to western-blotting 72 h after transfection (top) and protein expression was quantified by densitometry (bottom) using GAPDH as loading control for normalization. Relative quantification of Annexin A2 expression levels was graphically represented as Mean ± SEM for two independent experiments for panel a and three for panel b and c (* *p* < 0.05, ** *p* < 0.01).

In order to understand the meaning of the interaction between Hakai, Annexin A2 and Hsp90, first, we decided to confirm the previous proteomic study on which Annexin A2 was downregulated in stably Hakai-overexpressing MDCK cells compared to normal cells [21]. On one hand, it is reported that Hakai interacts with Src tyrosine-phosphorylated substrates, an on the other hand, Annexin A2 is tyrosine-phosphorylated by Src kinase [12,27]. We carried out a transient transfection of Flag-Hakai together with Src and HA-ubiquitin in HEK293T cells to favor the activity of the ubiquitin-mediated degradation system. Besides, co-overexpression of Hakai with Src favors the increase of Hakai levels [12,16]. As expected, Annexin A2 protein expression was significantly reduced by Hakai overexpression, while Hsp90 protein expression was not affected (Figure 3a, Supplementary Figure S6). Moreover, silencing Hakai protein levels by transiently transfecting two different Hakai siRNAs statistically significant increased Annexin A2 expression in HEK293T (Figure 3b, Supplementary Figure S6) and in HCT116 cells (Figure 3c, Supplementary Figure S6), without affecting Hsp90 protein expression. Moreover, this upregulation of Annexin A2 in HEK293T cell line was accompanied with an increase protein levels of the best-described substrate for the E3 ubiquitin-ligase Hakai, E-cadherin [12,16]. These data suggest that Hakai may act as an E3 ubiquitin-ligase for Annexin A2 protein, inducing its degradation.

### 2.4. Hsp90 Inbibitor Geldanamycin Induces Downregulation of Hakai Protein via Lysosome

**Figure 4:**
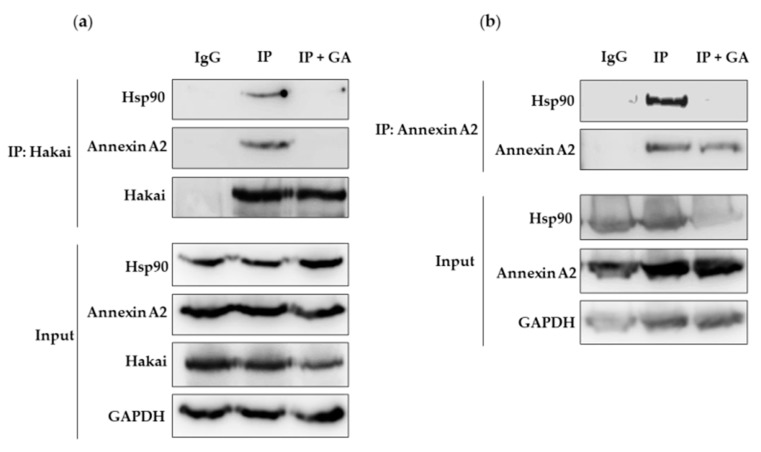
Geldanamycin treatment disrupts Hakai, Hsp90 and Annexin A2 interaction: HCT116 cell line was treated with 10 µM geldanamycin for 24 h. (**a**) Immunoprecipitation was performed by using Hakai antibody. Subsequent western blotting-analysis was performed using Hakai, Annexin A2, or Hsp90 antibodies. (**b**) Immunoprecipitation was performed by using Annexin A2 antibody and western blotting analysis was performed using Hakai and Hsp90 antibodies. GAPDH was used as protein loading control.

**Figure 5:**
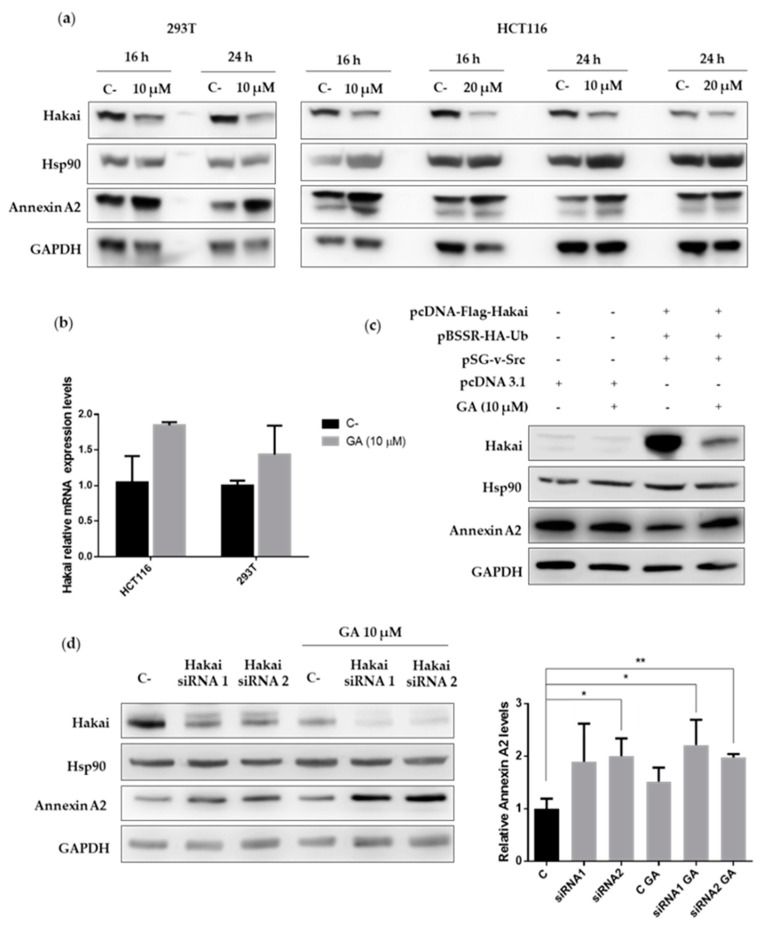
Geldanamycin reduces Hakai protein expression accompanied with an increase of Annexin A2: (**a**) HEK293T and HCT116 cells were treated with geldanamycin at the indicated times and concentrations. Cell lysates were collected and subjected to western blot analysis with the indicated antibodies. (**b**) HCT116 and HEK293T cell lines were treated with geldanamycin for 24 h at the indicated concentration. Cells were collected and subjected to mRNA extraction followed by RT-qPCR. Levels of Hakai mRNA normalized to control (HPRT) mRNA were measured and graphically represented as Mean ± SEM for three independent experiments (* *p* < 0.05, ** *p* < 0.01). (**c**) HEK293T cells were transiently transfected with pcDNA 3.1 (10 µg) empty vector or pcDNA-Flag-Hakai (4 µg), pBSSR-HA-ubiquitin (3 µg) and pSG-v-Src (3 µg) plasmids for 48 h. Cells were treated with 10 µM geldanamycin 24 h after transfection. Cells were collected and protein levels were evaluated by western-blotting with the indicated antibodies. (**d**) HCT116 cells were transfected with Hakai siRNA-1 and siRNA-2 oligos for 72 h and treated with 10 µM geldanamycin for the last 24 h of transfection. Cell lysates were collected and protein expression was evaluated by western-blotting with the indicated antibodies. Relative quantification of Annexin A2 expression levels was graphically represented as Mean ± SEM for three independent experiments (* *p* < 0.05, ** *p* < 0.01).

Geldanamycin is probably the best described Hsp90 inhibitor so far and acts by blocking its ATP binding site, preventing the correct folding of the client proteins that, in consequence, often turns into the degradation through proteasome [28,29]. Given the previously detected interaction between Hsp90, Hakai, and Annexin A2, we decided to study whether geldanamycin may affect these protein interactions. As shown, the described interaction between Hakai, Hsp90 and Annexin A2 was completely disrupted in presence of geldanamycin when using Hakai or Annexin A2 antibodies for the immunoprecipitation assays (Figure 4, Supplementary Figure S7). Given that Annexin A2 is proposed as a potential new substrate for the E3 ubiquitin-ligase Hakai and that geldanamycin disrupts the interaction between Hakai and Hsp90, it is open the possibility that Hakai might be a direct client protein for Hsp90 chaperone. In order to test whether geldanamycin could downregulate Hakai, we used two different concentrations of geldanamycin Hsp90 inhibitor (10 µM and 20 µM) and we treat two different cell lines, HEK293T and HCT116 cells for 16 and 24 h. The cells were collected and subjected to western-blotting analysis. As shown, geldanamycin treatment decreases Hakai protein levels in both cell lines tested, while an increase of Annexin A2 was detected (Figure 5a, Supplementary Figure S8). Furthermore, we found that treatment with geldanamycin did not decrease Hakai mRNA levels supporting that Hsp90 inhibitor downregulates Hakai at the post-transcription level (Figure 5b, Supplementary Figure S8). Moreover, we tested the effect of Hsp90 inhibitor on the downregulation of Hakai by transiently transfecting Flag-Hakai together with v-Src, and HA-ubiquitin in HEK293T cells. Hakai levels were drastically reduced in presence of geldanamycin compared to non-treated control transfected conditions, accompanied by an increase of Annexin A2 protein levels (Figure 5c, Supplementary Figure S8). These data confirm that geldanamycin is able to downregulate both endogenous and ectopically expressed Hakai while it upregulates Annexin A2. Finally, the effect on Hakai and Annexin A2 protein levels was tested by combining geldanamycin treatment together with two different siRNA Hakai oligos. HCT116 cells were transiently transfected with the indicated siRNA Hakai oligos for 72 h and treated with 10 µM geldanamycin for 24 h. Reduction of Hakai expression levels was accentuated when combining geldanamycin treatment together with the previously tested siRNA Hakai oligos, leading to almost Hakai completely disappearance (Figure 5d, Supplementary Figure S8). On the contrary, Annexin A2 levels were significantly increased. Altogether, these data demonstrate that geldanamycin Hsp90 inhibitor induce Hakai protein downregulation via post-transcriptional mechanism and support that Annexin A2 is a new substrate for the E3 ubiquitin-ligase Hakai protein.

**Figure 6:**
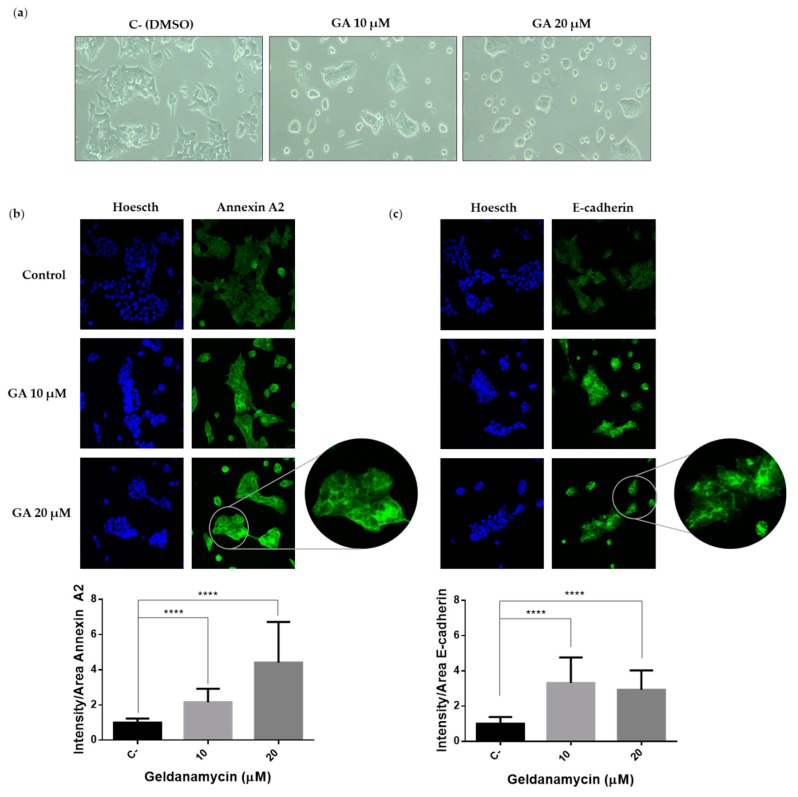
Geldanamycin induces an epithelial-like phenotype accompanied by an increase expression of E-cadherin and Annexin A2: HCT116 cell line was treated at the indicated concentrations of geldanamycin (GA) for 24 h. (**a**) Images of HCT116 were taken by optical microscopy. (**b**) Representative immunofluorescence images of Annexin A2 are shown (upper panel) and fluorescence intensity quantification is represented (lower panel). (**c**) Representative immunofluorescence images of E-cadherin are shown (upper panel) and fluorescence intensity quantification is represented (lower panel). Images were taken with confocal microscope by employing 40× magnification objective. A zoom image of 80X magnification was included. Annexin A2 and E-cadherin were stained in green, and cell nuclei were counterstained with Hoechst. Quantification of intensity/area was represented as Mean ± SEM (**** *p* < 0.0001).

As previously mentioned, Hakai is an E3 ubiquitin-ligase for E-cadherin that plays a role on the epithelial–mesenchymal transition program. Given the effect of geldanamycin on Hakai expression, we also analyzed the effect of geldanamycin on the cell phenotype. HCT116 epithelial cells were treated for 24 h with geldanamycin with the indicated concentrations showing a more epithelial phenotype under the treatment. Indeed, HCT116 loses the mesenchymal phenotype accompanied by decreasing cellular protrusions (Figure 6a). Moreover, we also analyzed the localization of Annexin A2 and E-cadherin by immunofluorescence. Annexin A2 expression was increased in presence of the geldanamycin showing a statistically enriched pattern in cytoplasm and cell membrane (Figure 6b).

On the other hand, we also observed a three-fold increase expression of E-cadherin at cell–cell contacts in presence of geldanamycin (Figure 6c). All these data support that Hakai is downregulated by geldanamycin inhibitor, which in consequence may influence the upregulation of E-cadherin and Annexin A2 proteins, and further reinforce the hypothesis of Hakai being an Hsp90 client protein.

**Figure 7:**
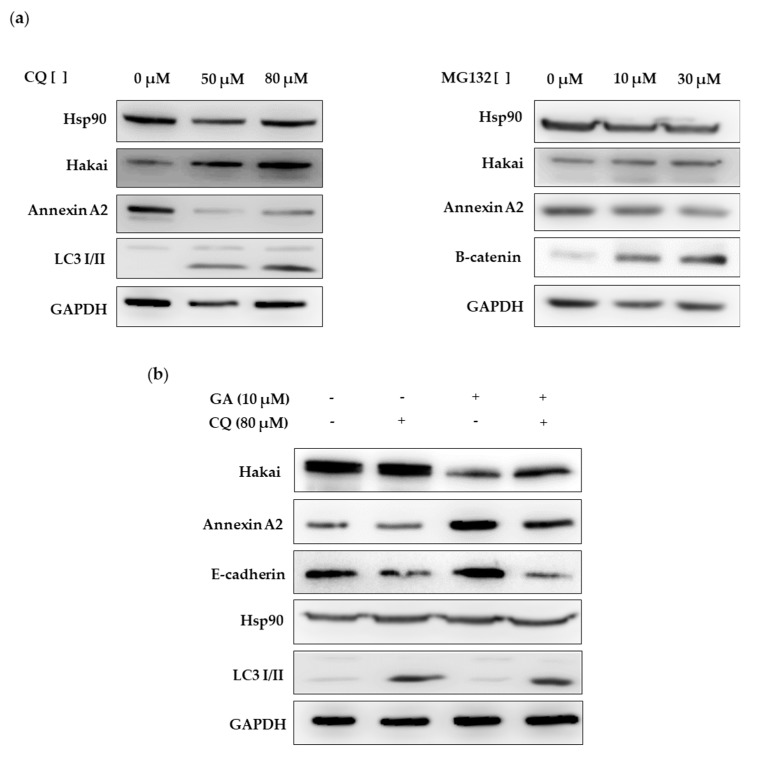
Geldanamycin increases Hakai protein degradation in a lysosome-dependent manner: (**a**) HEK293T cells were treated with chloroquine (left panel) and with MG132 (right panel) for 24 h at the indicated concentrations. Cell lysates were collected and evaluated by western blot analyses with Hakai and Annexin A2 antibodies. (**b**) HEK293T cells were transiently transfected with pcDNA-Flag-Hakai (4 µg), pBSSR-HA-Ubiquitin (3 µg) and pSG-v-Src (3 µg) for 48 h. The day after transfection, cells were treated with chloroquine and geldanamycin at the indicated concentrations for 24 h. Cell lysates were collected and protein expression was evaluated by western blot analyses using the indicated antibodies. Chloroquine treatment-induced Hakai protein levels recovery during geldanamycin treatment. LC3 I/II levels was used as a positive control in chloroquine treatment and β-catenin as positive control in MG132 treatment.

Then, we further investigated the possible mechanism of Hakai degradation under geldanamycin treatment. First, we analyzed the effect on Hakai protein expression of proteasome inhibitor MG132 and the lysosome inhibitor chloroquine (CQ). As shown, Hakai protein expression was increased in presence of chloroquine while no effect was observed in presence of MG132 (Figure 7a, Supplementary Figure S9), further indicating that Hakai may be degraded in a lysosome-dependent manner. Besides, Hakai levels increase was accompanied by a downregulation of Annexin A2, supporting the previously obtained results that suggest that Annexin A2 is as a new possible target protein for Hakai E3 ubiquitin-ligase. Next, we analyzed the mechanism by which Hakai is degraded in absence of Hsp90 function. In order to better detect Hakai downregulation under geldanamycin treatment, HEK293T cells were transiently transfected with Hakai, Src and Ubiquitin for 48 h and treated in presence or absence of geldanamycin and chloroquine for 24 h and protein lysates were analyzed by western blot. As shown, chloroquine lysosome inhibitor efficiently prevented Hakai degradation induced by geldanamycin (Figure 7b, Supplementary Figure S9). Accordingly, Annexin A2 was upregulated under geldanamycin treatment while this effect was reverted in combination with chloroquine inhibitor. Moreover, the well-described substrate for the E3 ubiquitin-ligase Hakai, E-cadherin, was also regulated in a similar manner than Annexin A2. All these data support that Hsp90 inhibitor geldanamycin induces downregulation of its client protein Hakai in a lysosome-dependent manner. Moreover, Hakai substrate E-cadherin was also regulated in a similar manner than Annexin A2. All these data support that Hsp90 inhibitor geldanamycin induces downregulation of its client protein Hakai in a lysosome-dependent manner. Moreover, E-cadherin and Annexin A2, were also affected by geldanamycin treatment, suggesting the involvement of Hsp90 chaperone in the regulation of Hakai specific substrates.

### 2.5. Downregulation of Hakai May Partially Account for the Pharmacological Anti-Migratory Effect of Geldanamycin Hsp90 Inhibitor

**Figure 8:**
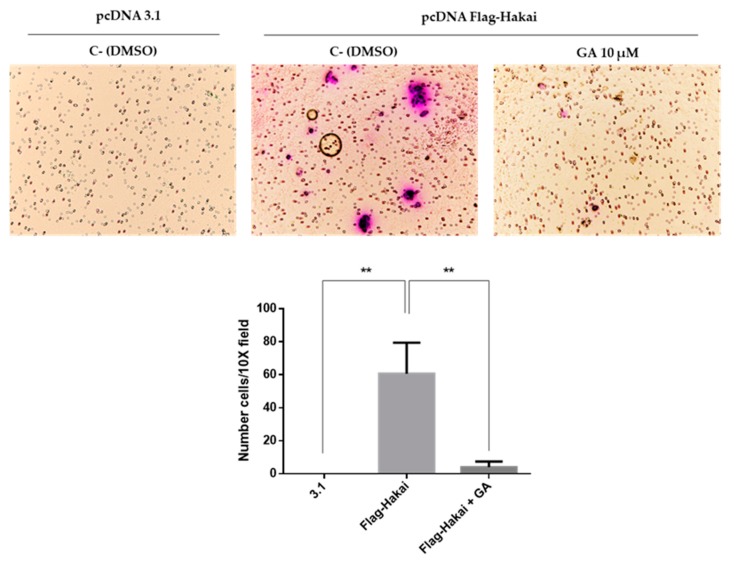
Geldanamycin reduces Hakai-induced cell migration: HEK293T cells were transiently transfected with 8 µg of empty vector pcDNA 3.1 or pcDNA-Flag-Hakai for 54 h and motility was evaluated by transwell migration assay in presence or absence of 10 µM geldanamycin. Cells were seeded into migration transwells 48 h after transfection and let to migrate for 16 h in a gradient concentration of FBS (1–30%). Then cells were fixed and stained as indicated in methods. Images were obtained by optical microscopy with an objective of 10X magnification. Representative images taken using the 10× objective are shown (**upper panel**) and quantification of migrating cells is shown (**bottom panel**). Results are represented as as Mean ± SEM of triplicates (** *p* < 0.01).

HSP90 is required for the stability and function of numerous oncogenic proteins, and its specific inhibitors display multiple anticancer effects [30,31,32]. On the other hand, Hakai is considered an oncogenic protein that was reported to be overexpressed in various cancers such as colon and lung cancer [16,19,20], furthermore, Hakai knockdown inhibits cell migration [33]. Therefore, we decided to test whether Hsp90 geldanamycin inhibitor may indeed influence the migratory effect driven by Hakai. Transwell migration assay was performed in HEK293T by transiently transfected with pcDNA 3.1 or pcDNA-Flag-Hakai. HEK293T cells transfected with pcDNA-Flag-Hakai would strongly increase cell migration compared to cells transfected with an empty vector (Figure 8). This migratory capability induced by Hakai overexpression was drastically reduced in presence of geldanamycin. herefore, our results support that geldanamycin treatment affects Hakai-mediated cell migration by reducing Hsp90 activity and consequently affecting Hakai-induced migration capacity.

### 2.6. Hsp90 Is Highly Expressed in Colorectal Cancer Samples Compared to Adjacent Normal Epithelial Tissues

**Figure 9:**
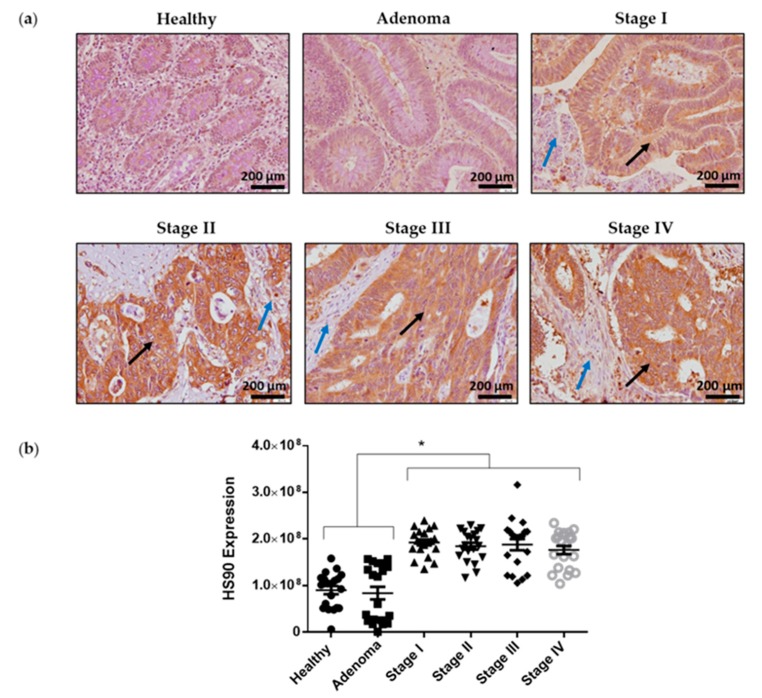
Hsp90 expression levels in colorectal cancer human samples: (**a**) Representative images of Hsp90 immunoreactivity in normal colonic mucosa, adenoma and colorectal cancer (TNM stages I–IV). Images were obtained with 20× objective. Scale bar 125 µm. (**b**) Statistical quantification of Hsp90 staining intensity in epithelial cancer cells at different colon cancer stages and in adenoma and normal colon tissue. A total of 30 human colon samples were analysed including: different TNM stages (I–IV) from colon adenocarcinomas, colon adenoma compared to human colon healthy tissues (normal colonic mucosa, n = 5; adenoma, n = 5; colorectal cancer, n = 5 of all stages) Scale bar, 200 µm. Black arrows show tumoral tissue and blue arrows show stromal tissue. Five photographs of each tissue were quantified and data are represented as scatter plot. Results are represented as Mean ± SEM for signal intensity scoring per area. Analysis was performed by employing Kruskal-Wallis with Tukey correction test (* *p* < 0.05).

The results described thus far suggest a link between Hsp90 and Hakai using an in vitro model. We previously demonstrated that Hakai expression levels are correlated to colorectal tumor progression, being proposed as a novel biomarker for colon cancer progression. Indeed, Hakai expression is gradually increased according to clinical TNM Classification System from UICC in adenoma and in different TNM stages (I–IV) from colon adenocarcinomas compared to human healthy colon tissues [20]. In order to ascertain whether Hsp90 might be involved in colorectal cancer in vivo, we investigated Hsp90 expression in human colorectal cancer patient samples. We performed immunohistochemistry experiments in samples of colorectal cancer patients, including healthy tissue, adenoma, and TNM stages I to IV of colorectal cancer. Hsp90 expression, but not Hsp70 (Supplementary Figure S3), is highly increased in carcinoma samples (TNM stage I–IV) compared to healthy epithelial tissue and adenoma (Figure 9). The changes in Hsp90 expression during colorectal cancer progression further support a pathological role for Hsp90 during tumor progression. Taken together, our studies reveal that Hsp90 is a critical regulator of Hakai protein expression and we propose that its influence on Hakai-regulated genes may, at least partially, impact in tumor progression.

## 3. Discussion

Hsp90 chaperone has been widely described to be implicated in cancer progression by regulating client proteins described as hallmarks in cancer disease. Most of the Hsp90 client proteins are implicated in cellular processes such as regulators of cellular proliferation, proteins of oxidative stress or proteins implicated in cellular differentiation between others [2]. Interestingly, a recent study show that more than a hundred E3 ubiquitin-ligases interact with Hsp90 and therefore many of them awaits to be elucidated [1,9]. In this study, we have shown that the E3 ubiquitin-ligase Hakai is a novel Hsp90-interacting protein (Figure 1). It is generally established that the interaction between Hsp90 and their client proteins results on the degradation of the clients by the ubiquitin-dependent proteasome pathway [5,6,7]. Indeed, pharmacological inhibition of the folding activity of Hsp90 is coupled to the action of different E3 ubiquitin-ligases, such as Cullin-5 and CHIP, that signal for ubiquitinization and degradation of the specific Hsp90 client proteins that have not been correctly folded [22,34]. Although this is the best-described mechanism, we demonstrate that Hakai is a novel client protein for Hsp90 (Figure 5). So far UHRF1 was the only reported E3 ubiquitin-ligase described as a client protein for Hsp90 [35]. Our results underscore that pharmacological inhibition of Hsp90 by geldanamycin results in the degradation of Hakai in a lysosome-dependent manner (Figure 7). A major challenge for future study is to identify the exact ubiquitin-ligase that mediates Hakai degradation upon pharmacological inactivation of Hsp90. To our knowledge, this proteasome-independent mechanism of degradation under Hsp90 geldanamycin inhibition was only previously described for IκB kinase. In this work, authors demonstrate that Hsp90 client protein IκB kinase (IkK), an essential activator of NF-kB, is degraded by autophagy when inhibiting Hsp90 with geldamacyn [8].

In previous work from our lab, we demonstrated that Annexin A2 is downregulated in Hakai-overexpressing MDCK epithelial cells compared to non-transformed MDCK cells [21]. Moreover, it has been previously shown an interaction between Annexin A2 and Hsp90 in vitro an in vivo in diabetic rat’s aorta [25]. Based on these reported results, and given the interaction confirmed between Hsp90 and Hakai in different cell lines, we were also interested in determining the possible relationship between Annexin A2, Hakai and Hsp90. As shown in Figure 2, by immunoprecipitation we confirmed an endogenous interaction between Hakai, Hsp90 and Annexin A2 in HCT116 cell line (Figure 2). Annexin A2 belongs to annexin family and is involved in the dynamic organization of membrane microdomains and the formation of membrane-cytoskeleton and membrane-membrane contacts [36]. Annexin A2 is tyrosine-phosphorylated by Src kinase [27] and, as previously mentioned, Hakai interacts with Src tyrosine-phosphorylated substrates inducing their ubiquitination and degradation, turning Annexin A2 into a potential substrate for Hakai. Transient overexpression of Hakai, Src and ubiquitin significantly downregulates Annexin A2 expression levels, and Hakai silencing give rise to an upregulation of endogenous Annexin A2 expression (Figure 3). These data confirm the direct regulation of Annexin A2 by Hakai, and suggest Annexin A2 could be a novel substrate protein for its E3 ubiquitin-ligase activity. Furthermore, the inhibition of Hsp90 with geldanamycin completely disrupted the interaction between Hakai, Hsp90 and Annexin A2 (Figure 4), while a decrease Hakai expression and an increase of Annexin A2 expression is observed. Moreover, a recovery of an epithelial phenotype in HCT116 cell line after geldanamycin treatment was observed, accompanied by an increase of Annexin A2 in cell membrane and E-cadherin at cell–cell contacts. Finally, geldanamycin reduces Hakai-induced cell migration. All these data support that the effect of the inhibition of Hsp90 by geldanamycin regulates Hakai client protein stability and, in consequence, E-cadherin Hakai-substrate and Annexin A2 are increased, which may partially account for the pharmacological the anti-migratory and the reversion of the EMT. This data are according to previously reported results on which ganetespib treatment or HSP90 knockdown downregulated molecular pathways associated with EMT and motility [37].

Despite the broadly described role of Annexin A2 as a poor-prognosis marker during cancer progression, it has recently been reported to be induced for degradation by other oncogenic E3 ubiquitin-ligases, such as TRIM65 [38,39]. Furthermore, Annexin A2, involved in the membrane-cytoskeleton dynamics, was demonstrated to play a role in the recovery of E-cadherin at adherens junctions due to its effect on actin cytoskeleton [40], further supporting the above-explained phenotype observed during geldanamycin treatment. On the other hand, it was shown that geldanamycin stabilizes E-cadherin through the degradation of Hsp90 client protein ErbB2, responsible of increasing β-catenin-E-cadherin association and thus, contributing to the maintenance of the epithelial phenotype [41]. These results suggest that geldanamycin might revert EMT process at least partially due to its effect on the stability of Hakai client protein of Hsp90, and in consequence supports Hakai effect on E-cadherin substrate and Annexin A2.

In this study, we evaluated the expression of Hsp90 in different colon adenocarcinoma stages in order to find an implication of this chaperone during tumor progression. Previous findings of our group show that Hakai expression is increased during colon cancer progression and it is considered as a novel biomarker for colon cancer development [20]. We show an increased expression of Hsp90 in human colorectal cancer samples compared to adenoma and to adjacent healthy tissue but no differences were detected between TNM stages I to IV, further suggesting the role of Hsp90 in the acquisition of tumor malignancy in colon cancer. This data are according to the correlation between Hsp90 expression and poor outcome in patients with colorectal cancer [42]. Given that Hakai oncogene is also aberrantly highly expressed in colorectal cancer [10,13,20,43], and the demonstrated interaction between Hsp90 and Hakai in vitro, future investigations on the role of Hsp90 and Hakai in vivo await to be elucidated. Promoting the degradation of Hsp90 oncogenic client proteins by inhibiting Hsp90 is considered as a promising new anticancer strategy [44,45]. Since the appearance of the first generation Hsp90 inhibitors, such as geldanamycin and radicicol, many derivative compounds have been developed and studied for cancer treatment and subjected to clinical trials [29,46]. Although Hsp90 inhibition has been widely studied for the treatment of different types of cancer, Hsp90 inhibition-based monotherapy has not yet reached the expected results due to different resistance mechanisms. So far, for colorectal cancer only combined therapies using Hsp90 inhibitors with chemotherapeutic agents have been successful [47]. Given that one of the drug resistance mechanisms is based on an induced EMT, the development of Hakai inhibitors together with Hsp90 inhibitors could be an attractive strategy for therapeutic interventions.

## 4. Materials and Methods

### 4.1. Plasmids, Antibodies, and Inhibitors

pcDNA-Flag-Hakai, pBSSR-HA-Ubiquitin, and pSG-v-Src were previously described (Fujita et al., 2002), pcDNA 3.1, pEGFP-C1 and pEGFP-Hakai were provided by Dr. Fujita (Institute for Genetic Medicine, Hokkaido University, Japan), and pcDNA-Flag-HA-Hsp90 was kindly provided by Dr. Paweł Bieganowski (Mossakowski Medical Research Centre Polish Academy of Sciences, Poland). For western blot Hakai antibody (36-2800, Invitrogen, Carlsbad, CA, USA) was used at 1:1000. Hsp90 antibody (sc-13119, Santa Cruz, Dallas, TX, USA) was used at 1:1000. Annexin A2 antibody (sc-28385, Santa Cruz, TX, USA) was used at 1:500. LC3 A/B antibody (4108, Cell Signaling, Leiden, The Netherlands) was employed at 1:1000. E-cadherin antibody (610182, BD Trans Lab, Franklin Lakes, NJ, USA) was employed at 1:1000. β-catenin antibody (Cell Signaling, Leiden, The Netherlands) was employed at 1:1000. GAPDH antibody (39-8600, Invitrogene) was used at 1:10,000. Mouse and rabbit secondary antibodies (NA934V, NA931V, GE Healthcare, Chicago, IL, USA) were used at 1:10,000. For immunofluorescence Hakai and Hsp90 antibodies were used at 1:100 concentration, Annexin A2 antibody (sc-47696, Santa Cruz, Dallas, TX, USA) was used at 1:50 and E-cadherin antibody (610182, BD Trans Lab, Franklin Lakes, NJ, USA) was used at 1:100. Alexa fluor 568-conjugated and Alexa fluor 488-conjugated antibodies (A10042, A11001, Thermofisher, Whaltham, MA, USA) were used at a concentration of 1:200. For immunoprecipitation, Hakai antibody (A302-969A, Bethyl, Montgomery, TX, USA), Hakai-2498 (provided by Yasuyuki Fujita), Annexin A2 (sc-47696, Santa Cruz, Dallas, TX, USA) and Hsp90 (sc-28385, Santa Cruz, Dallas, TX, USA) Protein A-Agarose (sc-2001, Santa Cruz, Dallas, TX, USA) Protein G PLUS-Agarose (sc-2002, Santa Cruz, Dallas, TX, USA), normal mouse IgG (sc-2025, Santa Cruz, Dallas, TX, USA) and normal rabbit IgG (A300-289A, Bethyl, Montgomery, TX, USA) were used.

For immunohistochemistry Hsp90 antibody (ab13492, Abcam, Cambridge, UK) was used at 1:500. Proteasome inhibitor MG132 (Sigma-Aldrich, St. Louis, MO, USA) was added for 6 h using 10 µM and 30 µM. Lysosome degradation inhibitor Chloroquine (c-6628, Sigma-Aldrich, St. Louis, MO, USA), proteasome inhibitor MG132 (M8699, Sigma-Aldrich, St. Louis, MO, USA) and HSP90 Geldanamycin inhibitor (T6343, TargetMol, Wellesley Hills, MA, USA) were employed as indicated in figure legends.

For Hsp90 chaperone activity, HEK293T and HCT116 were incubated with geldanamycin at the indicated times and concentrations. Lysosome degradation inhibition was performed by incubating cells with chloroquine for 24 h at the indicated concentrations. Proteasome degradation inhibition was performed by incubating cells with MG132 for 6 h at the indicated concentrations.

### 4.2. Cell Culture and Transfection

HEK293T, HCT116, and ACHN cell lines were cultured in Dulbecco’s Modified Eagle’s Medium (DMEM) (Gibco, Thermofisher). HT29 cell line was cultured in McCoy’s Modified medium and LoVo cell line was cultured in F-12K medium (Kaighn’s Modification of Ham’s F-12 Medium) (Gibco, Thermo Fisher Scientific). All media were supplemented with 10% heat-inactivated fetal bovine serum (FBS) and 1% penicillin/streptomycin. All cell lines were grown at 37°C in a humidified incubator with 5% of CO2. All cell lines were periodically tested for mycoplasma. Transfection experiments were performed by employing Lipofectamine 2000 Transfection Reagent (Thermo Fisher Scientific) and Opti-MEM (Thermo Fisher Scientific) media following manufacturer’s protocol. Transfection was performed for 24 or 48 as indicated in figure legends.

### 4.3. Western Blot Analysis and Immunoprecipitation

For protein extraction, cells were lysed employing 1% Triton X-100 buffer (20 mM Tris-HCl pH 7.5, 150 mM NaCl and 1% Triton X-100) supplemented with 1 mM phenylmethylsulfonyl fluoride and 1× leupeptin/aprotinin mix. Lysis was performed for 30 min at 4 °C and centrifuged at 14,000× *g* for 10 min. Protein quantification was performed by employing Pierce BCA Protein Assay Kit (Thermo Fisher Scientific) following manufacturer’s indications. Twenty micrograms of protein sample were loaded in polyacrylamide gels. Western blot protocol was performed as previously described (Cano et al., 2000). Densitometric quantification was performed by using ImageJ Software (National Institutes of Health, NIH). For immunoprecipitation, protein extracts from 100 mm dishes were incubated with 60 µL of Protein A Agarose Beads (for rabbit antibodies) or Protein G PLUS-Agarose (for mouse antibodies) (sc-2001, sc-2002, Santa Cruz, Dallas, TX, USA) for 1 h at 4 °C in rotation for pre-clearing. Hakai-2498 and commercial antibodies or control IgG were incubated onto 60 µL of beads in 500 µL of PBS-Tween 20 1% for 1 h in rotation at 4 °C. Beads were precipitated by centrifugation at 3000 rpm, 2 min at 4 °C and 80 µL of supernatants were collected for input total lysates. Supernatants were incubated for 2 h with beads-antibody complexes at 4 °C on rotation. Beads were washed twice with lysis buffer and samples were prepared for western blotting by adding 40 µL of Laemmli buffer to the immunoprecipitated sample and 20 µL to the input sample.

### 4.4. Human Samples and Immunohistochemistry

Human colorectal cancer samples were collected by Pathological Anatomy department of Complejo Hospitalario Universitario de A Coruña (CHUAC) under informed consent from the patients. Research Ethics Committee from A Coruña-Ferrol approved their use for investigation according to the standard ethical procedures described in the “Ley Orgánica de Investigación Biomédica” of 14 July 2007 of the Spanish regulation (ethical protocol code: 2015/024). Paraffin samples were transferred by CHUAC Biobank, included in the Spanish Hospital Platform Biobanks Network. For immunohistochemistry, slides containing sections of tumors were deparaffinized for 1 h at 60 °C in the stove. Once dewaxed, the slides were rehydrated by successive incubations in xylol (10 min), xylol (10 min), ethanol 100° (10 min), ethanol 96° (10 min), ethanol 70° (10 min) and water (5 min). Antigen retrieval was performed by boiling the slides for 15 min using target retrieval solution pH 6.1 (Agilent, Santa Clara, CA, USA). Slides were washed with PBS-T 1% for 10 min and peroxidase blocking solution (DakoCytomation, Glostrup, Denmark) was added for 30 min at RT. Slides were washed and carefully dried and blocked with blocking solution (0.2% BSA/0.1% Triton X-100 in PBS pH 7.6) for 30 min at RT. Incubation with primary antibody was carried out in a wet chamber overnight at 4 °C. Following primary antibody incubation, slides were washed three times for 10 min and incubated with secondary antibody (Dako REAL™ Envision™ Detection System). Slides were washed three times and revealed with diaminobenzidine (Dako REAL™ Envision™ Detection System) for 2 min. Slides were washed with water for 5 min and counterstained with Gill’s Hematoxylin for 20 s. Samples dehydratation was performed following the opposite sequence of alcohols for rehydratation. Samples were mounted with the coverslips using DePeX (Serva, Heidelberg, Germany). Pictures were obtained with Olympus microscope employing the objectives indicated in figure legends. Hsp90 postivity was evaluated based on brown-color developed by diaminobenzidine intensity.

### 4.5. Immunofluorescence Assays

Immunofluorescence was performed as previously described. Briefly were plated in sterile glass coverslips contained in 6-well plates washed twice with PBS-Tween 1%, fixed with PFA 4% for 15 min and permeabilized with 0.5% Triton X-100/PBS for 15 min. After permeabilization, cells were blocked for 1 h with culture medium supplemented with 10% FBS. Primary antibodies were incubated for 2 h at RT. Secondary antibody was incubated at RT for 1 h. Nuclear staining was performed by employing 1:10,000 Hoechst dilution (Life Technologies, Carlsbad, CA, USA). ProLong Gold Antifade Mountant (Life Technologies, Carlsbad, CA, USA) was employed for coverslips mounting. Images were obtained by using confocal microscope Nikon A1R. Immunofluoresce intensity was evaluated by employing ImageJ software (National Institutes of Health, NIH). Intensity of ten different areas for two different replicates were quantified and relativized against the mean control area. Values are the means ± SEM of the staining intensity signal scoring per area. Calibration and quantification of the images were performed with ImageJ software. Statistical analysis was carried out by using unpaired Student’s *t*-test at the indicated significance levels.

### 4.6. RNA Interference

Hakai silencing was performed by employing two different siRNA oligonucleotides: Hakai-1 (CTCGATCGGTCAGTCAGGAAA) and Hakai-2 (CACCGCGAACTCAAAGAACTA). Oligonucleotides were transfected into cells by using Lipofectamine 2000 Transfection reagent (Thermo Fisher Scientific). Transfection reaction was prepared by mixing 2 µL (200 pmol) of 100 µM oligonucleotides with 4 µL and proceeded as for plasmid transfection. Same quantity of Mission Universal Non-coding siRNA (Sigma-Aldrich, St. Louis, MO, USa) was used as a negative control of transfection. Cells were transfected for 72 h.

### 4.7. Real-Time Quantitative PCR (RT-qPCR)

HCT116 and HEK293T total RNA was extracted using TriPure isolation reagent (Roche, Basel, Switzerland). mRNA levels were analysed in technical triplicates by quantitative RT-PCR, following specifications of reverse retrotranscriptase kit (NZYTech, Lisbon, Portugal). Amplification was performed in a Light Cycler 480 (Roche, Basel, Switzerland) and data was analysed by qBase+ analysis software (Biogazelle, Zwijnaarde, Belgium). Primers used for Hakai were F’ TGCTATGACTGTGCATTTTACATGA and R’ ACTGCTAATTCGCTGCAC. HPRT was used as housekeeping using primers F’ TGACCTTGATTTATTTTGCATACC and R’ CGAGCAAGACGTTCAGTCCT.

### 4.8. Migration Assay

Cell migration assay was performed by employing 24-well Cell Migration Plate 8 µm (Merck Millipore, Burlington, MA, USA) HEK293T cells were transiently transfected for 48 h as indicated. Cells were starved 18 h prior to assay. After 48 h of transfection, cells were collected and seeded at a confluence of 3 × 10^5^ cells/transwell in a gradient of 1–30% FBS between upper and lower chamber respectively. Cells were let to migrate for 16 h and non-migratory cells were carefully removed from de upper chamber with a moistened cotton swap. Migratory cells were fixed for 20 min with 4% PFA, rinsed with PBS pH 7.4 and stained with crystal violet for 20 min. Finally, transwells were rinsed with PBS pH 7.4 and membrane was removed and mounted onto slides. Migratory cells were photographed with an Olympus BX50 employing a 10× objective.

### 4.9. Statistical Analysis

Analysis was performed by employing GraphPad Prism 6 Software (GraphPad Software Inc., San Diego, CA, USA). Western blotting statistical analysis was carried out by using unpaired Student’s *t*-test at the indicated significance levels. Graphical representations of results are expressed as Mean ± SEM. Statistical analysis for immunofluorescence was carried out by performing comparative student’s *t* test. Results are represented as Mean ± SEM for 10 areas of two different photographs. Migration assay quantification was performed by quantifying the number of migratory cells per 10× objective field for three different photographs. Statistical analysis was performed by employing Student’s *t*-test and represented as Mean ± SEM for three photographs of one experiment. Human IHQ quantification was performed by using Kruskal-Wallis with Tukey correction test. Significance of both Student’s *t*-test and Kruskal-Wallis with Tukey correction is indicated in the figures as * *p* < 0.05, ** *p* < 0.01 and *** *p* < 0.001.

## 5. Conclusions

In this study, we have demonstrated that the E3 ubiquitin-ligase Hakai is a novel client protein for Hsp90. Although the best-described mechanism for degradation of the Hsp90 clients proteins is through a ubiquitin-dependent proteasome pathway, we have shown that pharmacological inhibition of Hsp90 by geldanamycin results in the degradation of Hakai in a lysosome-dependent manner. Together with the disappearance of Hakai by geldanamycin treatment, a more epithelial phenotype was observed, accompanied by an increase expression of Hakai E-cadherin substrate at cell–cell contacts and Annexin A2 at plasma membrane. More importantly, geldanamycin reduces Hakai-induced cell migration, further underscoring the possible impact of Hsp90 inhibitors on EMT and tumor progression by its action on Hakai stability.

## Supplementary Materials

The following are available online at https://www.mdpi.com/2072-6694/12/1/215/s1, Figure S1: Concentration-dependent and kinetics assays for Hakai and Hsp90, Figure S2: Co-localization of Hsp90 and Hakai in different colon cancer cells, Figure S3: Hsp70 expression levels in human samples from colorectal cancer patients, Figure S4: Full blots corresponding to Figure 1, Figure S5: Full blots corresponding to Figure 2, Figure S6: Full blots corresponding to Figure 3, Figure S7: Full blots corresponding to Figure 4, Figure S8: Full blots corresponding to Figure 5, Figure S9: Full blots corresponding to Figure 7, Figure S10: Full blots corresponding to Supplementary Figure S1.
