# Supplementary material for: Heat Shock Protein 90 Chaperone Regulates the E3 Ubiquitin-Ligase Hakai Protein Stability
Source: Cancers (Basel). 2020 Jan 15;12(1):215. doi: 10.3390/cancers12010215 (PMC7017148; doi:10.3390/cancers12010215)
Supplement: Supplementary file 1 [file cancers-12-00215-s001.pdf]

cancers

Heat Shock Protein 90 Chaperone Regulates the E3 Ubiquitin-Ligase Hakai Protein Stability

Andrea Díaz-Díaz, Daniel Roca-Lema, Alba Casas-Pais, Gabriela Romay, Giovanni Colombo, Ángel Concha, Begoña Graña, and Angélica Figueroa

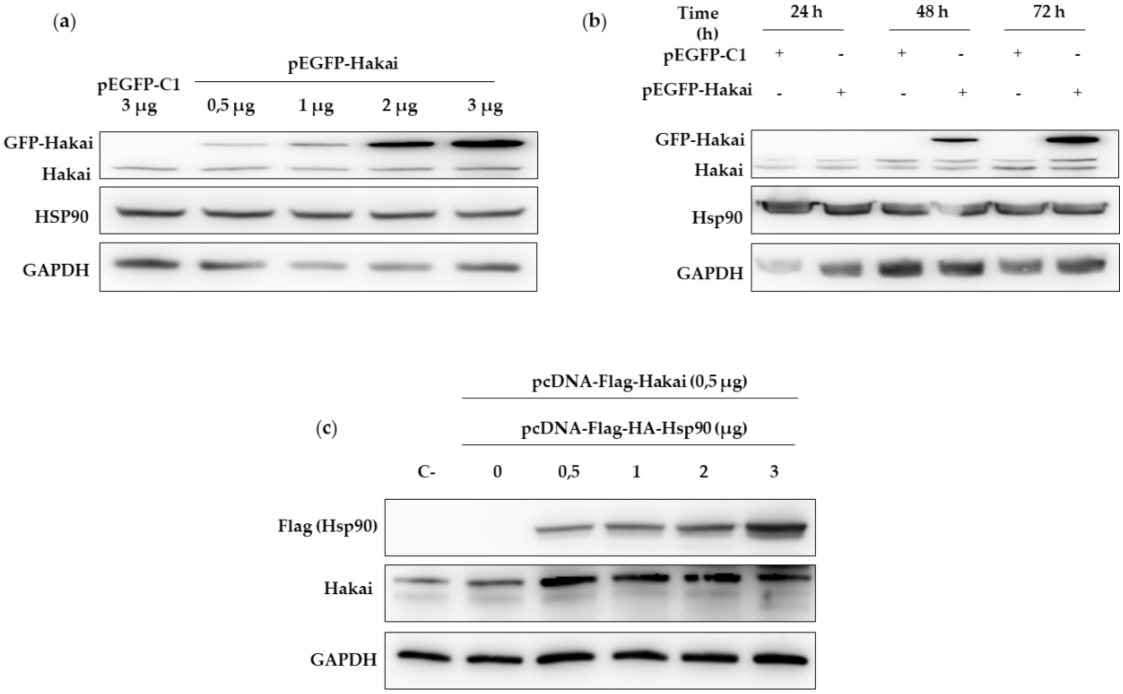

Figure S1. Hakai and Hsp90 overexpression do not affect their protein expression levels, respectively: (**a**) HEK293T cells were transfected for 48 h with increasing amounts of pEGFP-Hakai plasmid (0 -3 µg) completed with pEGFP-C1 empty vector until reaching maximum concentration of 3 µg. (**b**) HEK293T were transfected with 3 µg of pEGFP-Hakai and pEGFP-C1 empty vector and lysates were collected at different times of transfection until 72 h. (**c**) HEK293T cells were transfected for 48 h with 0.5 µg of pcDNA-Flag-Hakai and increasing amounts of pcDNA-Flag-HA-Hsp90 plasmid (0–3 µg). pcDNA 3.1 empty vector was used to complete until reaching maximum amount of 3 µg per point. Cell lysates were examined by western blot using the indicated antibodies.

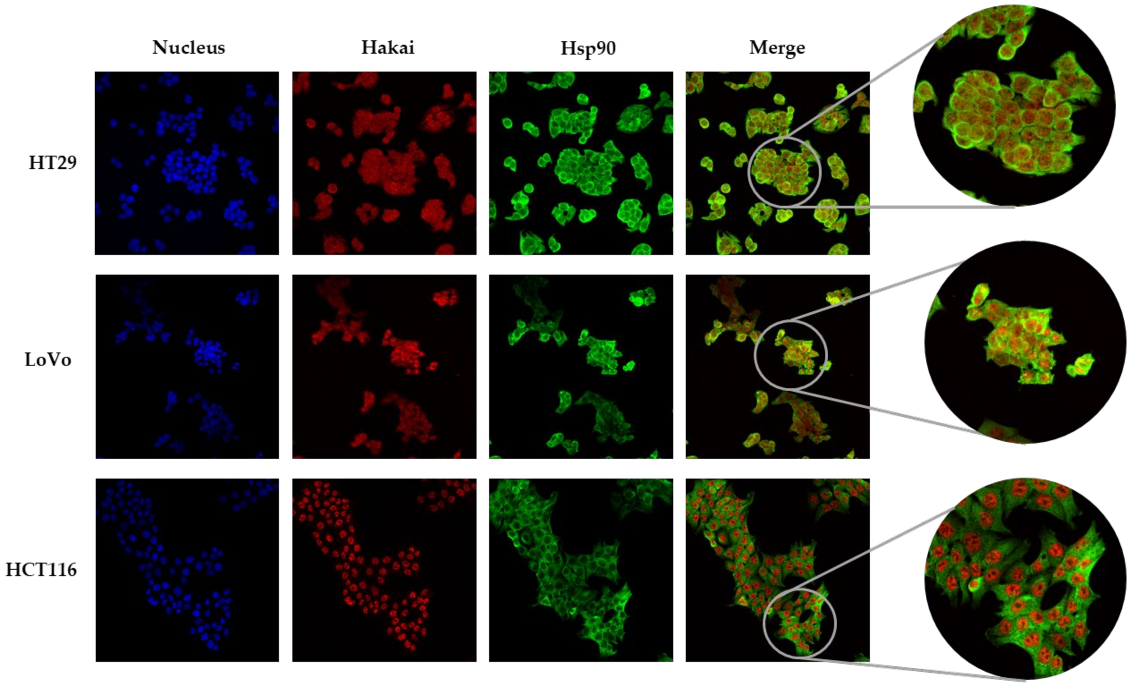

Figure S2. Hakai co-localization with Hsp90 in HT29, LoVo and HCT116 cell lines: Protein localization was examined by confocal microscopy. Slight co-localization between both proteins was detected in HT29 and LoVo cells in perinuclear areas. Photos were taken with an objective of 40X magnification. Zoom images were taken with 80X objective magnification.

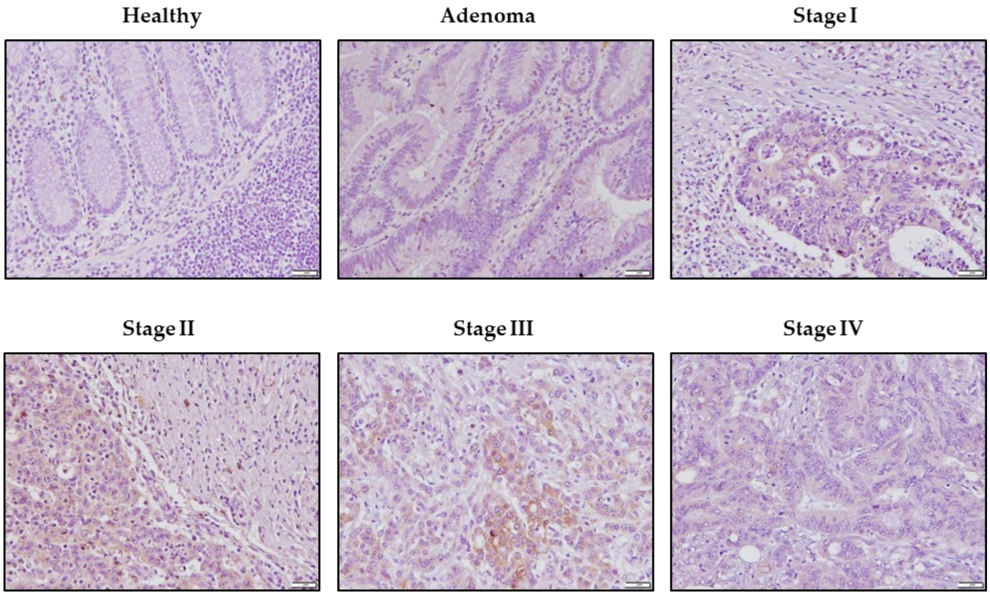

Figure S3. Hsp70 expression levels in colorectal cancer human samples: Representative images of Hsp70 immunoreactivity in healthy tissue, adenoma and TNM stages I–IV of colorectal cancer samples. Thirty human colon samples were analyzed including: different TNM stages (I-IV) from colon adenocarcinomas, colon adenoma and adjacent colon healthy tissues (normal colonic mucosa, n = 5; adenoma, n = 5; colorectal cancer, n = 5 of all stages). Immunohistochemistry protocol was performed as indicated for Hsp90. Images were obtained with 20× objective. Scale bar 125 µm.

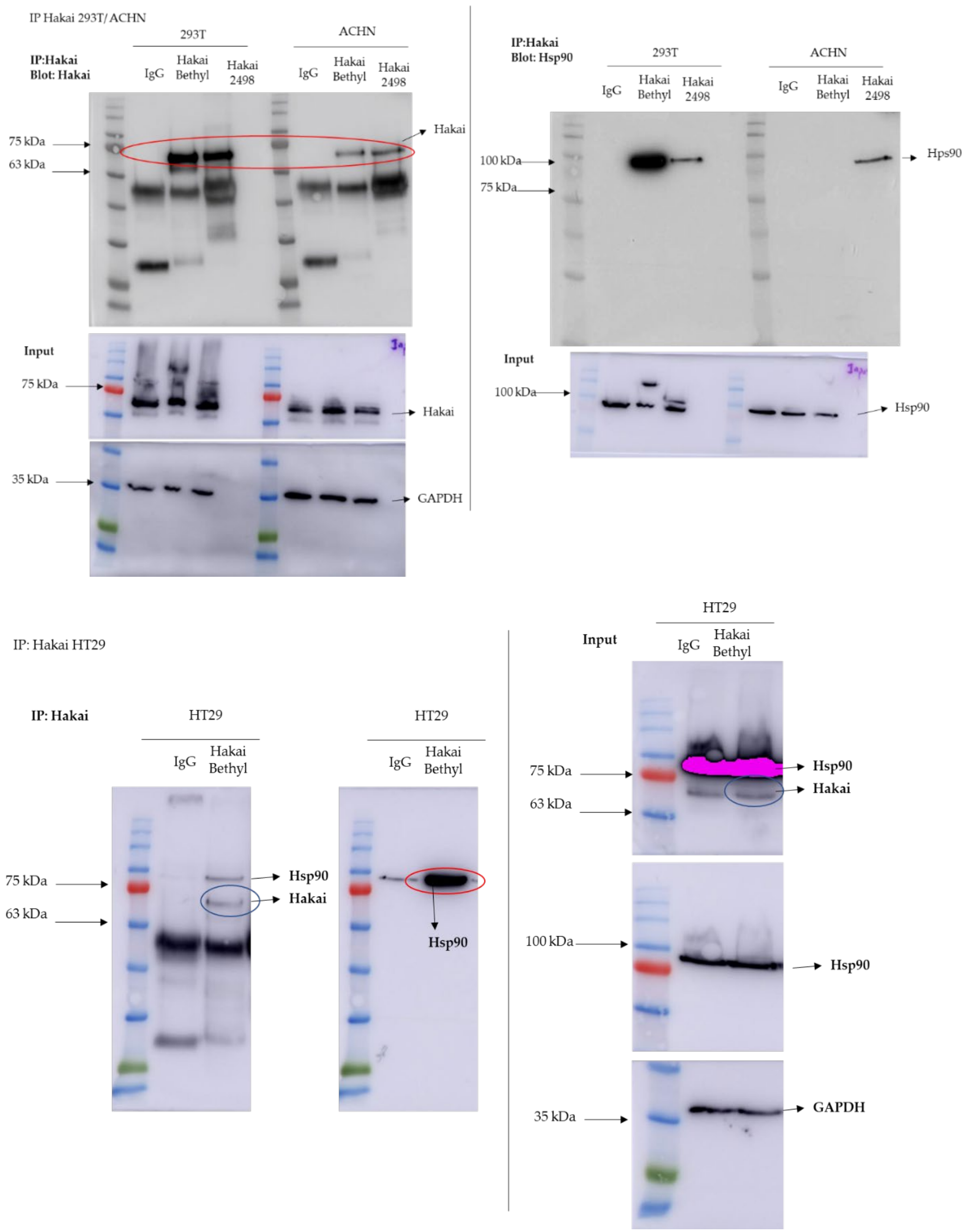
IP Hakai 293T/ACHN
293T
ACHN
IP:Hakai
Blot: Hakai
IgG
Hakai Bethyl
Hakai 2498
75 kDa
63 kDa
Hakai
Input
35 kDa
GAPDH
IP:Hakai
Blot: Hsp90
100 kDa
Hps90
Hsp90
IP: Hakai HT29
IP: Hakai
HT29
Input
Hsp90
Hakai

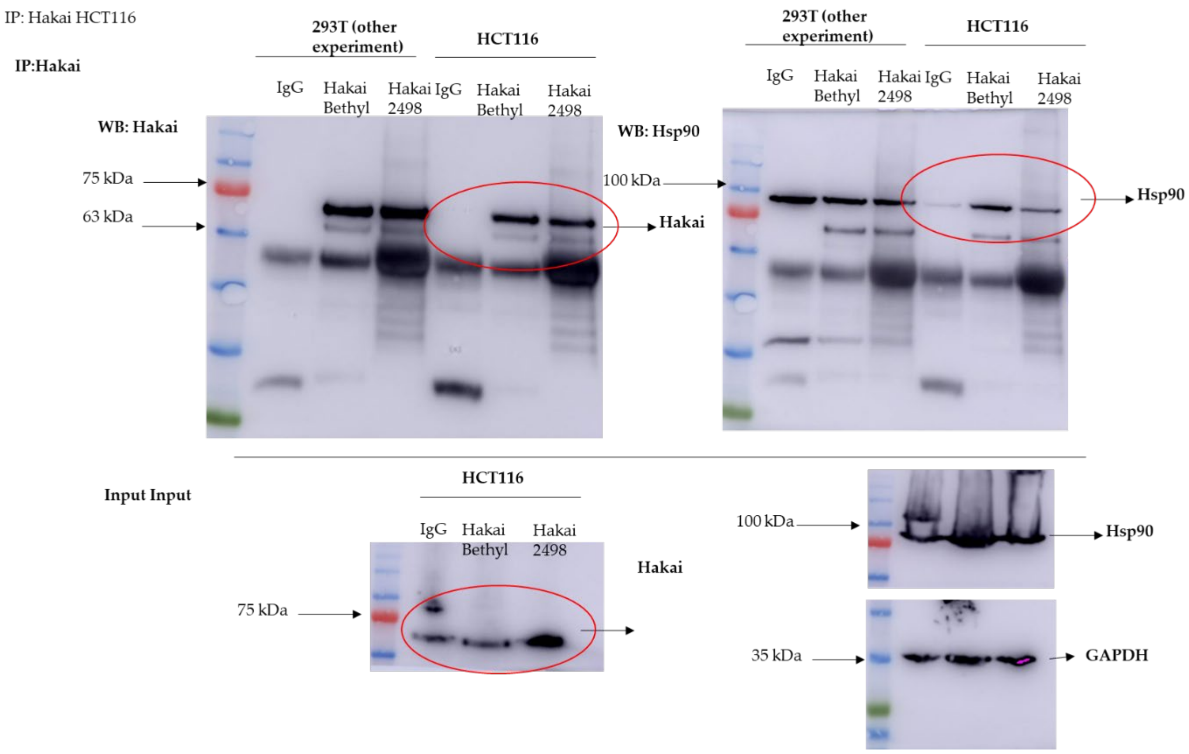

Figure S4. Full blots corresponding to Figure 1

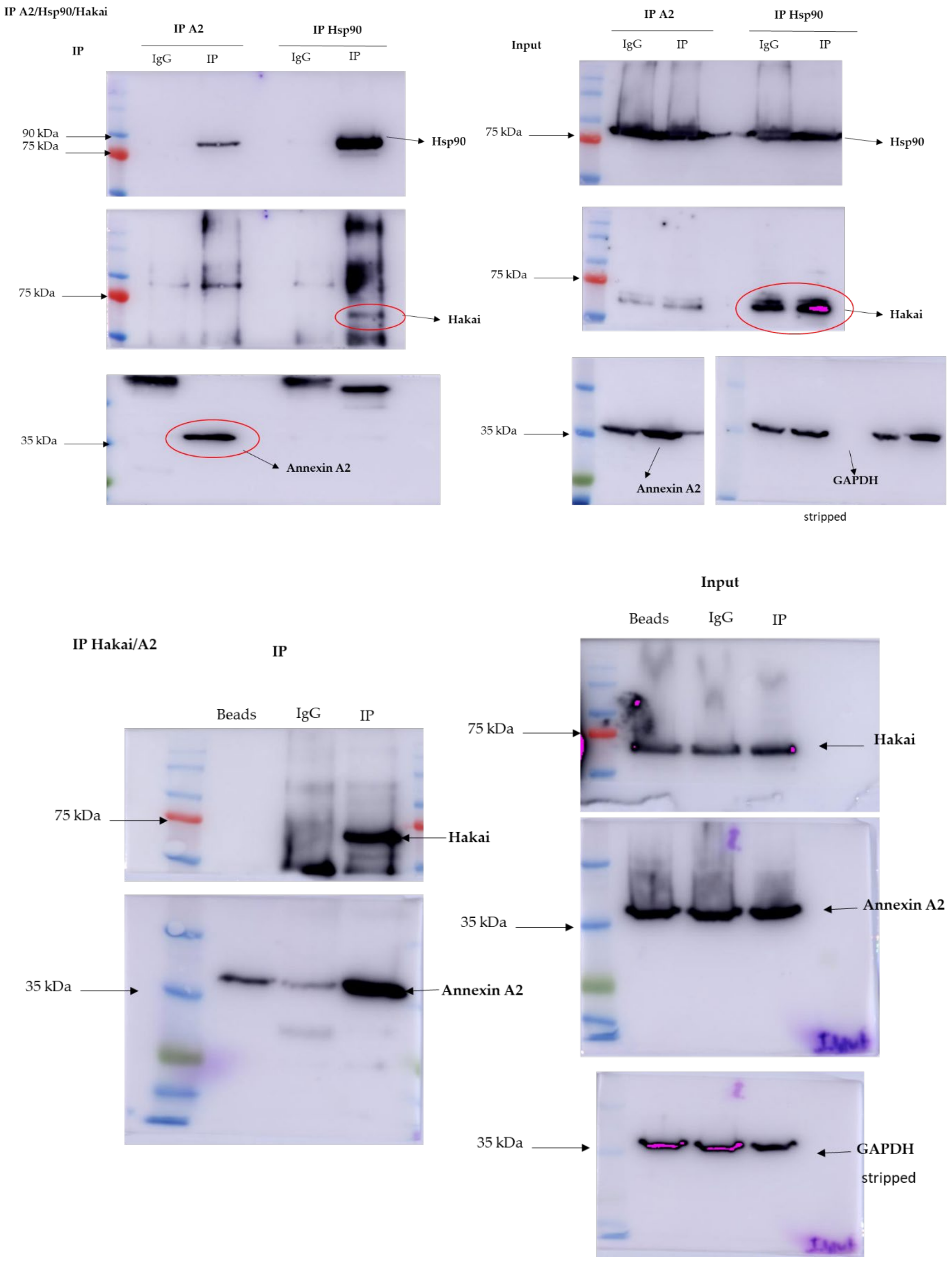

Figure S5. Full blots corresponding to Figure 2.

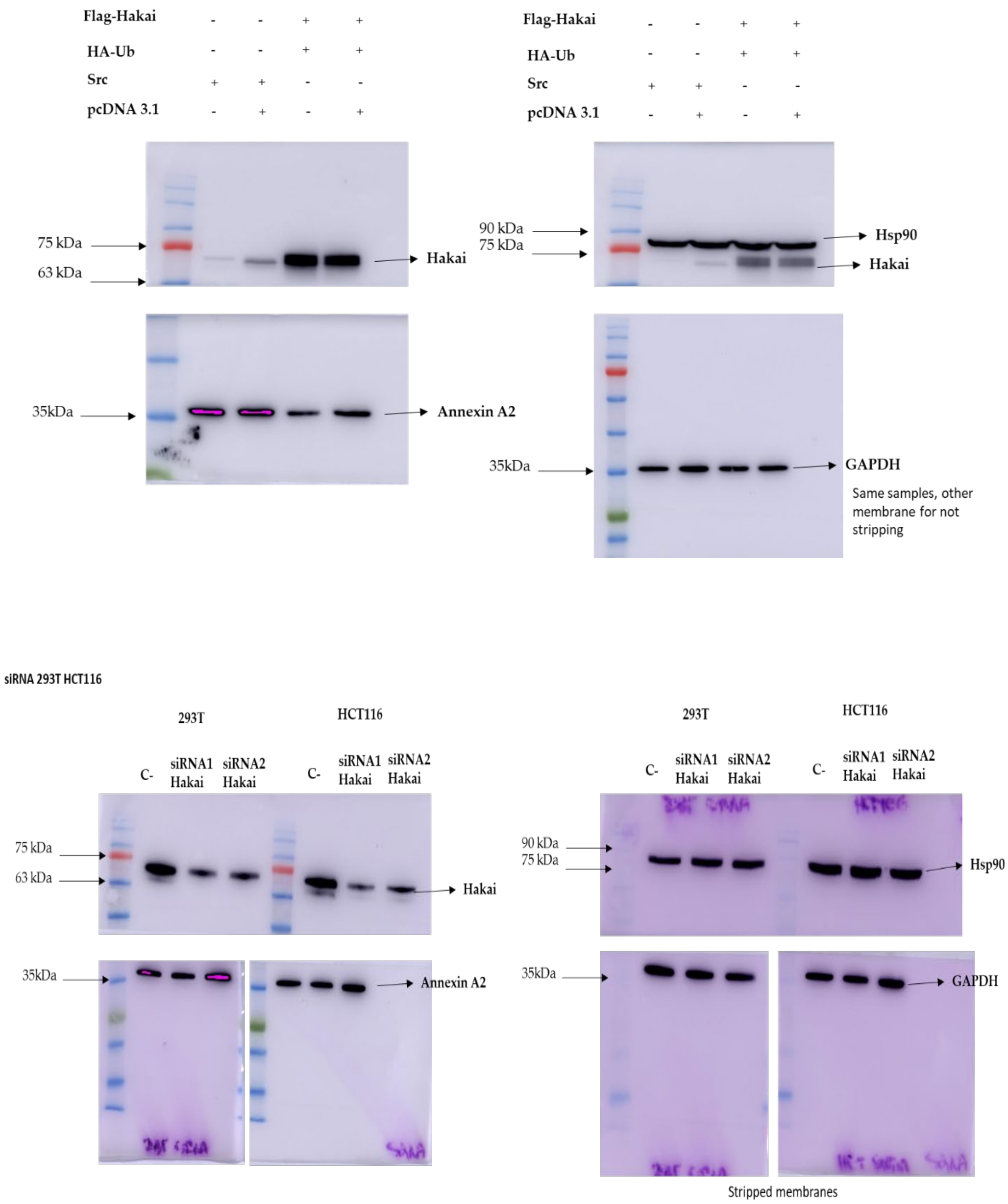

Figure S6. Full blots corresponding to Figure 3.

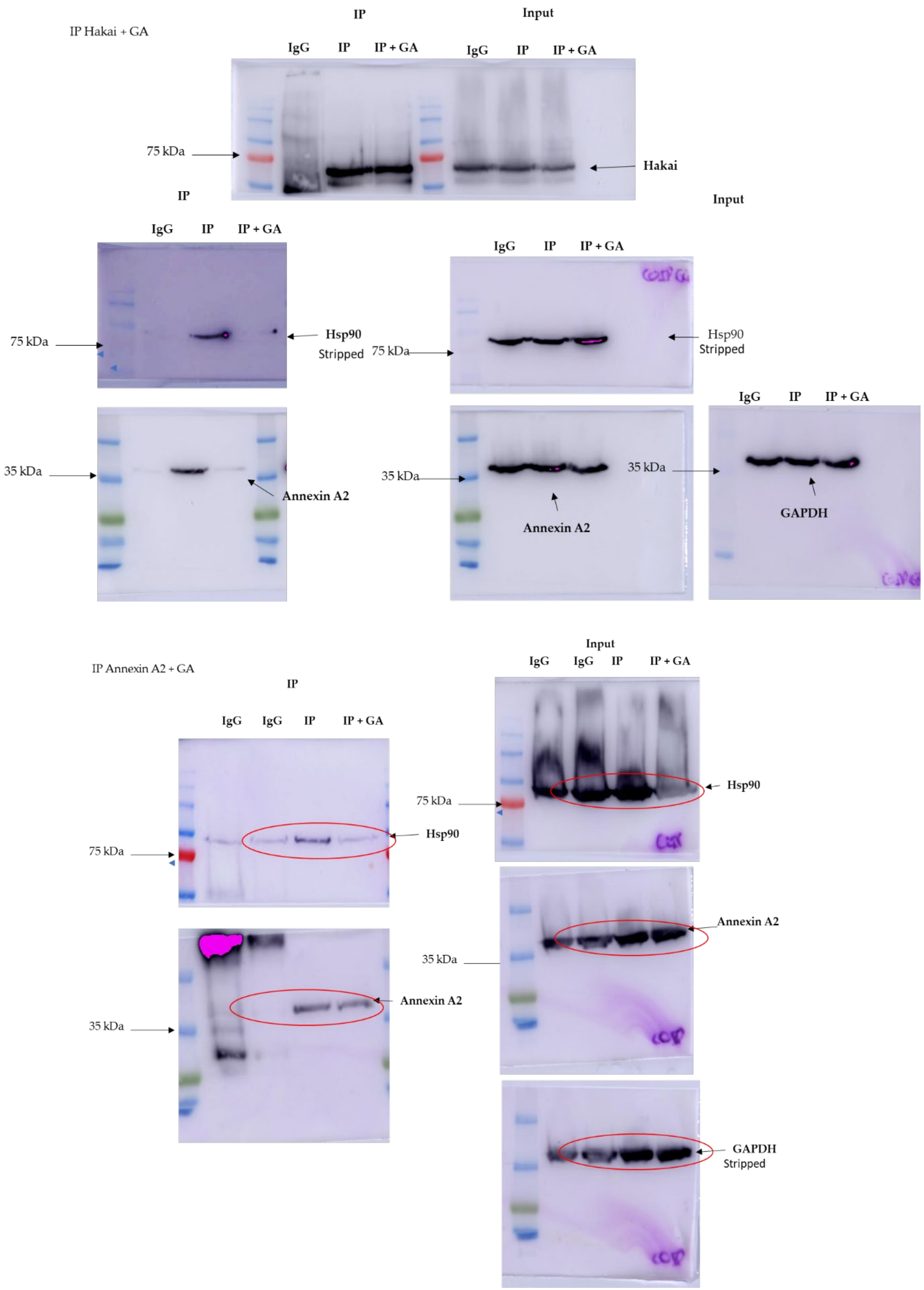

Figure S7. Full blots corresponding to Figure 4

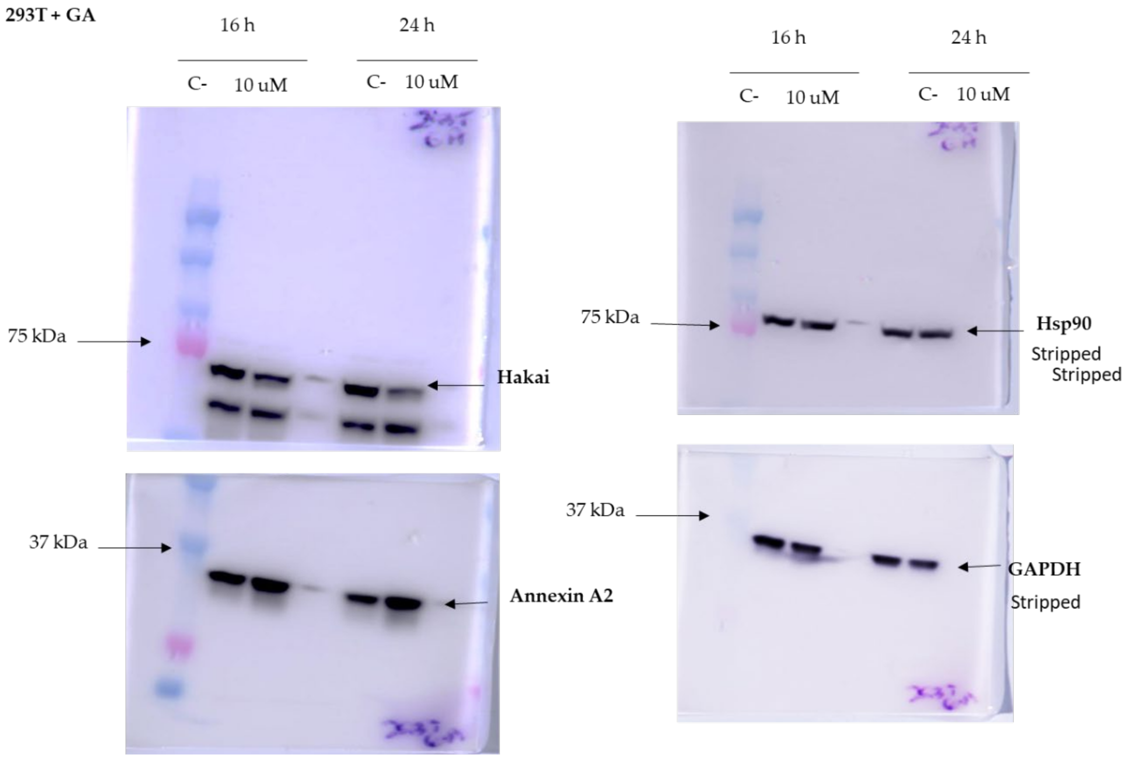
293T + GA
16 h
24 h
C-
10 uM
C-
10 uM
75 kDa
Hakai
37 kDa
Annexin A2
16 h
24 h
C-
10 uM
C-
10 uM
75 kDa
Hsp90
Stripped
Stripped
37 kDa
GAPDH
Stripped

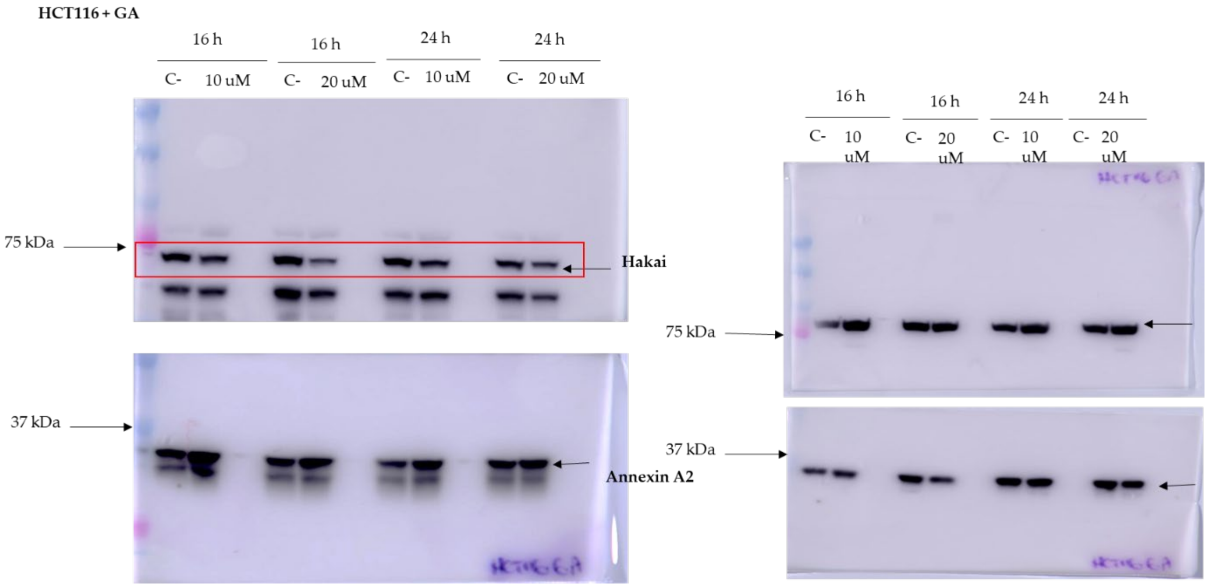
HCT116 + GA
16 h
16 h
24 h
24 h
C-
10 uM
C-
20 uM
C-
10 uM
C-
20 uM
75 kDa
Hakai
37 kDa
Annexin A2
16 h
16 h
24 h
24 h
C-
10 uM
C-
20 uM
C-
10 uM
C-
20 uM
75 kDa
37 kDa

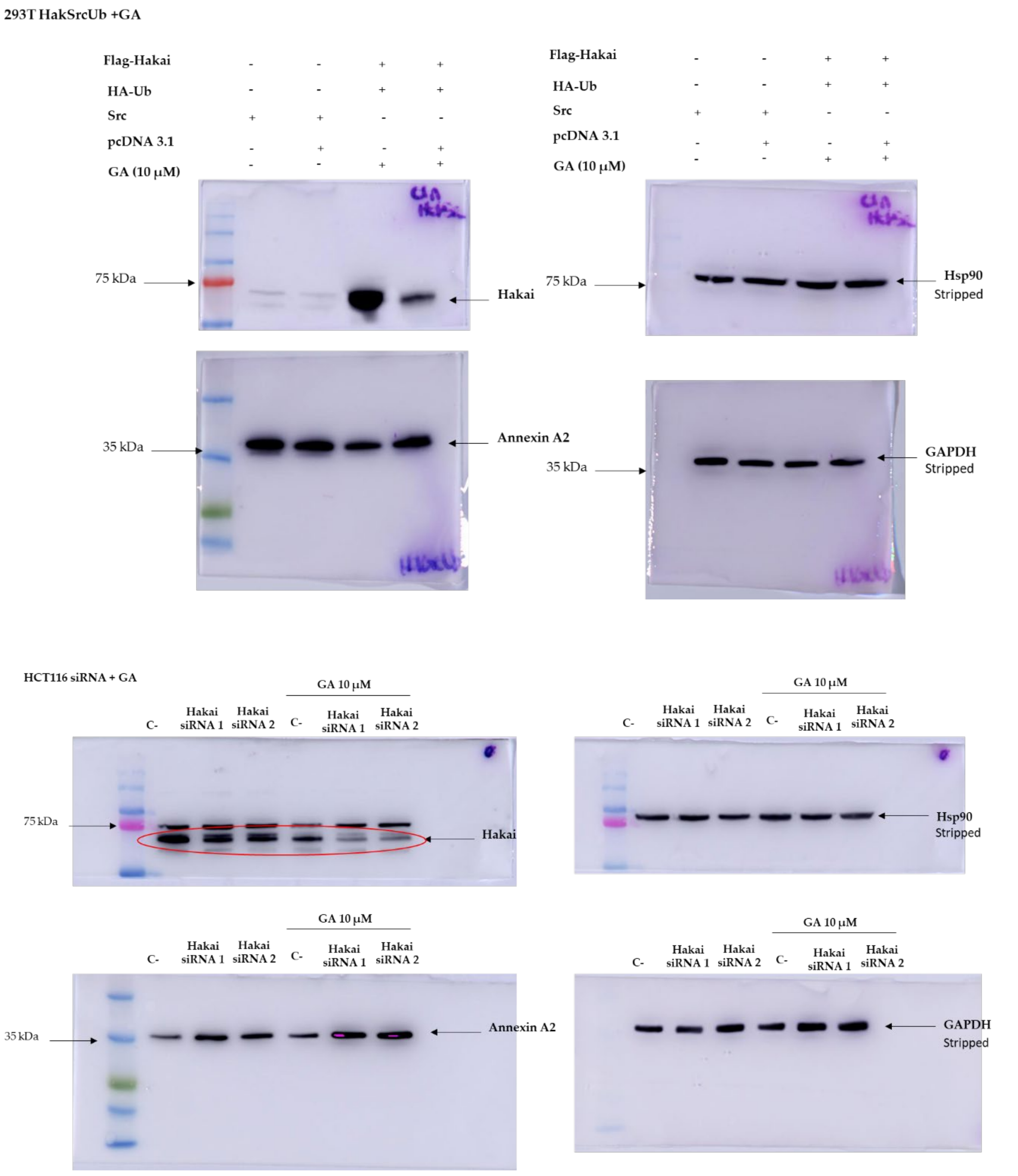

Figure S8. Full blots corresponding to Figure 5.

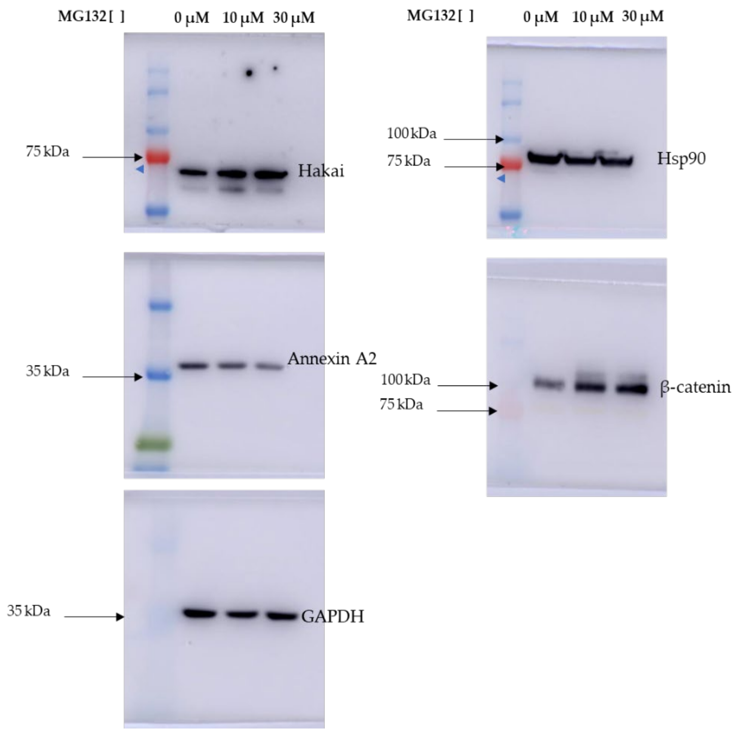

293T + CQ

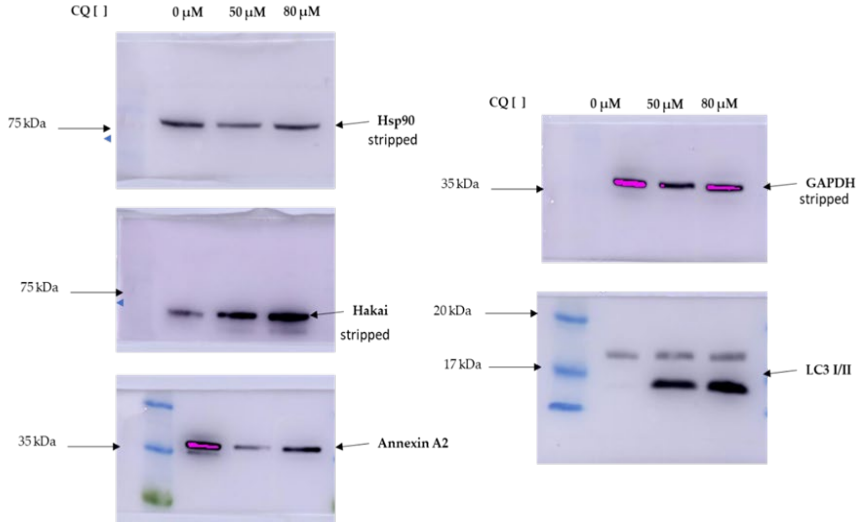

Hakai+ Src + Ub + CQ/GA

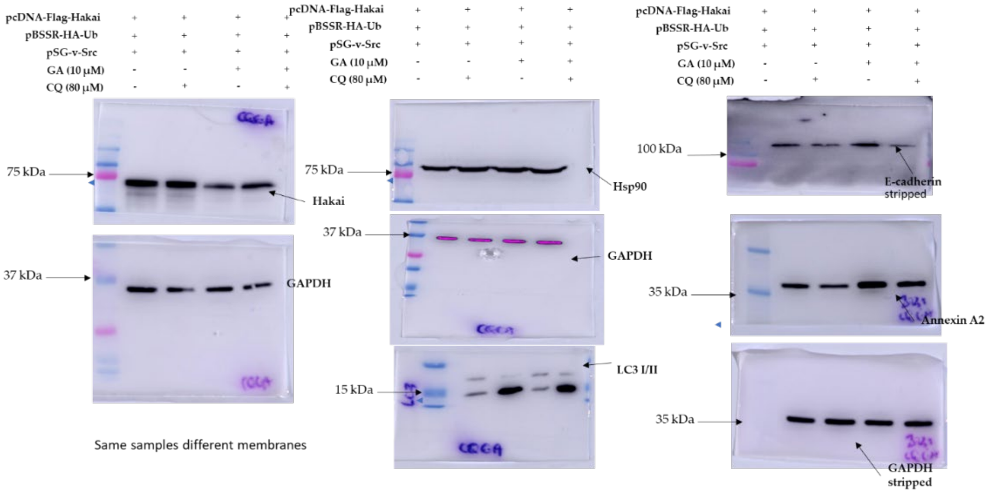

Figure S9. Full blots corresponding to Figure 7.

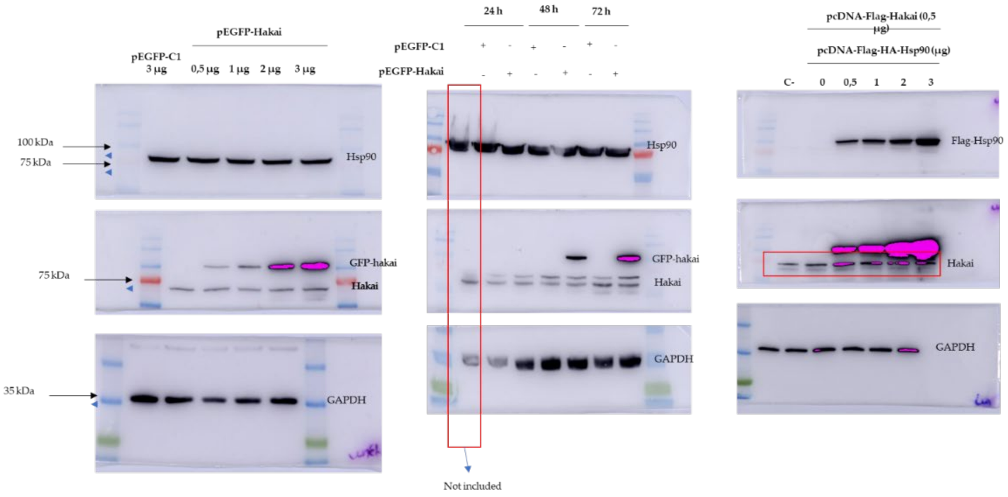

Figure S10. Full blots corresponding to Supplementary Figure S1.
